# Genome-Wide Identification and Characterization of the Xyloglucan Endotransglucosylase/Hydrolase (*XTH*) Gene Family in *Camellia oleifera* and the Function of *CoXTH1* During Drought Stress

**DOI:** 10.3390/plants14233605

**Published:** 2025-11-26

**Authors:** Yushen Ma, Ying Zhang, Zhen Zhang, Zhilong He, Chengfeng Xun, Xiangnan Wang, Yufeng Zhang, Rui Wang, Yongzhong Chen

**Affiliations:** 1 Research Institute of Oil Tea Camellia, Hunan Academy of Forestry, Changsha 410004, China; mys9204@163.com (Y.M.); hfazz@hnlky.cn (Z.Z.); patricezhang@163.com (Y.Z.); 2 National Engineering Research Center for Oil Tea Camellia, Changsha 410004, China; 3 Yuelushan Laboratory, Changsha 410004, China; 4 State Key Laboratory of Woody Oil Resources Utilization, Changsha 410004, China

**Keywords:** *CoXTHs*, drought tolerance, functional verification, hemicellulose, cellulose, cell wall structure

## Abstract

Xyloglucan endotransglucosylase/hydrolase (XTH) plays a significant role in plant responses and adaptation to abiotic stresses. However, the *XTH* gene family in *Camellia oleifera* remains largely unknown. Herein, 31 *CoXTH* genes from the *C. oleifera* genome, which were clustered into four evolutionary groups, were identified. Notably, *CoXTH1*, *CoXTH6*, *CoXTH14*, *CoXTH28*, and *CoXTH31* showed significant upregulation under drought stress, suggesting their importance in stress responses. Furthermore, heterologous expression of *CoXTH1*, *CoXTH14*, and *CoXTH28* in yeast improved yeast survival under drought stress. Overexpressing *CoXTH1* in *Arabidopsis thaliana* significantly enhanced drought tolerance, characterized by improved seedling growth, increased antioxidant enzyme activity, and reduced reactive oxygen species (ROS) levels. Notably, transgenic expression of *CoXTH1* significantly elevated the contents of xyloglucan, leading to increased cellulose, and hemicellulose contents in the plants. The elevated hemicellulose and cellulose strengthen the cell wall structure, maintaining cellular integrity and stability, and improving plant drought tolerance. These findings lay a foundation for understanding the functional roles of *CoXTH* genes and highlight *CoXTH1* as a potential candidate gene for improving drought tolerance in *C. oleifera* and other woody crops.

## 1. Introduction

Drought is among the most destructive and complex natural disasters globally, severely impacting plant growth and reducing crop yields [1,2,3,4]. Extreme drought can trigger food crises and famine, devastating human societies [5]. The global drought-induced crop losses over the past decade have totaled approximately USD 30 billion [6]. Human-induced global warming combined with meteorological phenomena, such as changes in precipitation, evaporation, and monsoon patterns, has intensified water distribution inequities, increasing drought frequency and severity, worldwide [7]. For instance, drought critically affects seedling survival and the forest establishment of *Camellia oleifera*, *C. oleifera* is extensively cultivated in the hilly regions of southern China and generally requires abundant water, light, and heat resources [8]. However, its growth and development are significantly limited by the mismatched spatial and temporal distribution of rainfall that worsens global warming, leading to frequent droughts [9].

Water-deficient plants can maintain cellular water homeostasis through a series of adaptive changes at the morphological, growth and developmental, photosynthetic, physiological, biochemical, metabolic, and molecular levels to mitigate the effects of drought stress [10]. The plant cell wall, which is the outermost cellular layer, and is primarily composed of cellulose, hemicellulose, pectin, and lignin [11], serves as a structural protective barrier and maintains cellular integrity by regulating osmotic pressure. Moreover, the cell wall is crucial in plant responses to drought stress and overall environmental adaptability [12]. The cell wall responds to drought stress through structural modifications, such as regulating its thickness, hardness, and extensibility, thereby enhancing drought resistance [13]. A cell wall-modifying enzyme, xyloglucan endotransglucosylase/hydrolase (XTH), belonging to the glycoside hydrolase 16 (GH16) subfamily, plays a crucial role in drought response [14]. XTH primarily catalyzes modifications to xyloglucan by cleaving and rejoining xyloglucan molecules, thereby modifying the cellulose–xyloglucan network within the cell wall [15]. XTH is also widely recognized as possessing two principal catalytic functions: xyloglucan endotransglucosylase (XET) activity and xyloglucan endohydrolase (XEH) activity [16].

The *XTH* gene family has been identified in a wide range of plant species. However, the number of *XTH* members varies across species. For example, *Oryza sativa* contains 29 family members [17], *Arabidopsis thaliana* contains 33 members [18], while *Populus trichocarpa* contains 41 members [19]. Moreover, researchers have classified these genes into four subgroups based on sequence similarity and phylogenetic relationships: Group I, II, IIIA, and IIIB. Members of Group I, II, and IIIB predominantly exhibit XET activity, while those in Group IIIA primarily display XEH activity [17]. The variation in enzymatic activities among different XTH family members suggests their diverse roles in modifying the cell wall structure.

Recent studies postulate that *XTH* genes are crucial in plant responses to drought stress. Esmaeilzadeh-Moridani et al. [20] reported that *OsXTH5* and *OsXTH19* are upregulated under drought stress and are highly expressed in drought-tolerant *O. sativa* varieties, suggesting their potential involvement in conferring drought tolerance. Similarly, overexpressing *DKXTH1* from *Diospyros kaki* increases cell wall density and promotes intercellular space development in transgenic *A. thaliana* plants, improving drought tolerance [21]. In transgenic *A. thaliana*, *ZmXTH30* enhances drought tolerance by reducing the accumulation of reactive oxygen species (ROS) and enhancing antioxidant enzyme activity [22]. Conversely, *TaXTH17* negatively regulates drought stress response in *Triticum aestivum* [23]. Similarly, overexpressing *HvXTH1* suppresses gene expression in the phenylpropanoid pathway involved in lignin biosynthesis, reducing stomatal closure and increasing plant susceptibility to drought stress [24]. These findings indicate that *XTH* genes are broadly implicated in plant drought responses, despite their significantly varied specific regulatory mechanisms and functional roles.

*C. oleifera* is a unique woody oilseed tree species native to China [8]. Its major production regions are in southern China, which are frequently affected by drought stress, attributed to the spatiotemporal uneven distribution of rainfall and the impact of summer-autumn droughts. Water scarcity has become a primary constraint on the development of the *C. oleifera* industry. Current studies on the drought tolerance of *C. oleifera*, particularly regarding its molecular response mechanisms to drought stress, remain relatively limited. Therefore, there is an urgent need to identify drought-resistant genes in *C. oleifera* and study the genetic basis of its drought tolerance. The *XTH* gene family plays a crucial role in plant responses to drought stress. However, the response characteristics and underlying molecular mechanisms of *XTH* family members in *C. oleifera* under drought stress remain largely unexplored. Herein, we hypothesized that the *XTH* gene family enhances drought tolerance in *C. oleifera* by modulating its cell wall compositions because it is a woody oil crop rich in lignocellulosic biomass. The *CoXTH* gene family was subsequently identified, followed by a comprehensive analysis of their gene structures, protein architectures, chromosome location, *cis*-acting regulatory elements, and expression patterns under drought stress. Moreover, a key candidate gene, *CoXTH1*, was selected for heterologous expression in *A. thaliana* to study the cell wall compositions related to drought tolerance. The results demonstrated that *CoXTH1* enhances cellular structural stability in drought-stressed plants by modulating cell wall remodeling, thereby improving drought tolerance. The findings of this study provide valuable genetic resources for enhancing drought resistance traits in woody oil crops, such as *C. oleifera*, and fill a critical knowledge gap associated with *XTH*-mediated drought response in woody oil crops.

## 2. Results

### 2.1. Identification of CoXTHs

**Figure 1:**
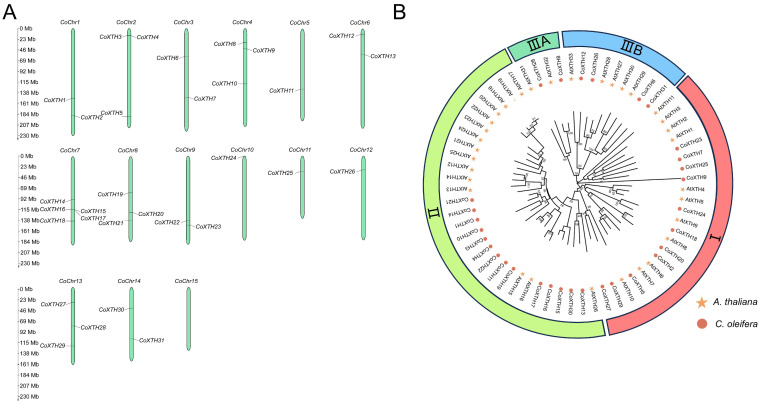
Specific characterization of *CoXTH* family genes and their encoded proteins. (**A**) Chromosomal locations of the *CoXTH* gene family; (**B**) Phylogenetic relationships of XTH proteins from *C. oleifera* and *A. thaliana*. The phylogenetic tree was generated using the Neighbor-Joining (NJ) method, with 1000 bootstrap replicates. Each color represents a specific group.

A total of 31 *CoXTH* genes were identified from the *C. oleifera* genome and sequentially named from *CoXTH1* to *CoXTH31* according to their chromosomal positions (Figure 1A). These genes were unevenly distributed across chromosomes, with *CoChr7* having the highest gene density, while CoChr15 had none. The chromosome length and number of *CoXTH* genes showed no significant positive correlation. The CoXTH proteins contained 256 to 352 amino acids, with isoelectric points (pI) ranging from 4.73 to 9.31 and their molecular weights from 28.63 to 40.14 kDa, which suggested that they were hydrophilic. Subcellular localization predictions indicated that most CoXTH proteins were localized in the cell wall, except for CoXTH1, CoXTH3, CoXTH11, CoXTH14, CoXTH17, CoXTH19, CoXTH21, CoXTH22, and CoXTH24, which were predicted to be located in the cytoplasm or cell wall (Table 1).

### 2.2. Phylogenetic Relationships, Gene Structure, and Conserved Motifs of CoXTHs

Figure 1B shows the phylogenetic relationships among CoXTH proteins from *C. oleifera* and AtXTH proteins from *A. thaliana*, which cluster the CoXTH proteins into four subgroups. Most proteins (25) were clustered in Groups I and II, while Group IIIA had the fewest proteins (CoXTH8 and CoXTH28). Gene structure analysis revealed high similarity within groups. Most genes had four introns, except for *CoXTH9* in Group I and members of Groups IIIA and IIIB, which had three introns. In Group II, *CoXTH20* had no introns, *CoXTH30* and *CoXTH13* had four introns, while the rest had three (Figure S1A). All CoXTH proteins contained ten conserved motifs (Motif 1–10) (Figure S1B), with detailed sequences in Table S1. Proteins within the same subgroup generally shared similar motif patterns despite some variations. Notably, all CoXTH proteins contained Motifs 5 and 6. Motif 5 was the crucial active site for catalytic activity (Figure S2), playing a critical role in gene function [25].

### 2.3. Synteny of CoXTHs

**Figure 2:**
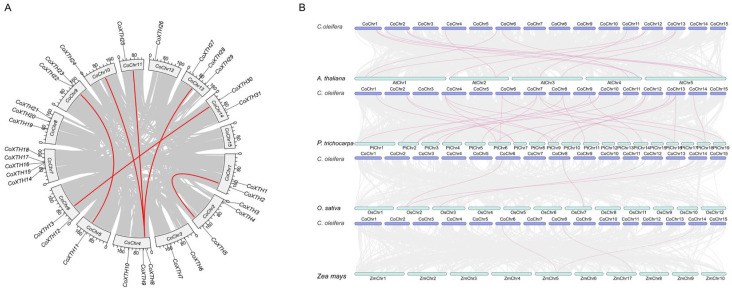
The collinearity of *CoXTH* family genes. (**A**) Intraspecific collinearity of *CoXTH* family genes. The red lines represent the gene duplication in *CoXTH* family genes; (**B**) Inter collinearity of XTH family genes in *C. oleifera*, *A. thaliana*, *O. sativa*, *P. trichocarpa*, and *Z. mays*. The gray lines indicate gene duplication between all genes in the different species during evolution. The red lines indicate gene duplication between the *CoXTH* and *XTH* genes of different species.

In the *CoXTH* gene family of *C. oleifera*, 4 gene pairs, *CoXTH22*-*CoXTH11*, *CoXTH8*-*CoXTH28*, *CoXTH30*-*CoXTH13*, and *CoXTH2*-*CoXTH5*, underwent segmental duplication events (Figure 2A). Therefore, tandem duplication may be the primary driving force in the expansion of the *CoXTH* gene family. Comparisons of the 31 *CoXTH* genes with the whole-genome sequences of *A. thaliana*, *O. sativa*, *P. trichocarpa*, and *Z. mays* revealed 18 homologous gene pairs between *C. oleifera* and *P. trichocarpa*, 13 between *C. oleifera* and *A. thaliana*, four between *C. oleifera* and *O. sativa*, and three between *C. oleifera* and *Z. mays* (Figure 2B; Table S2).

### 2.4. Cis-Acting Regulatory Elements of CoXTH Promoters

**Figure 3:**
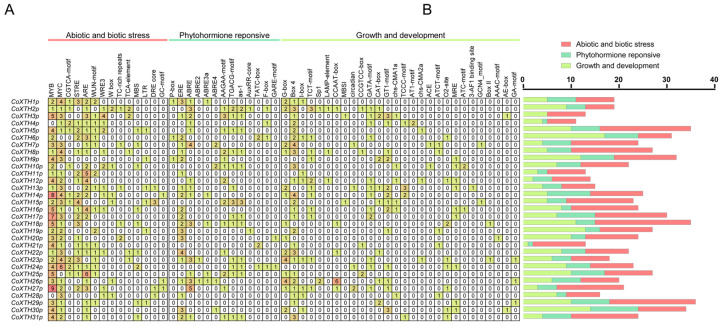
Promoter information of *CoXTH* family genes. (**A**) The number of *cis*-acting regulatory elements in the *CoXTH* promoter. The numbers in the boxes indicate the quantity of elements. The higher the intensity of the red color, the more elements in that number and vice versa; (**B**) The number of *cis*-regulatory elements that respond to abiotic and biotic stress, phytohormones, and growth and development.

Manual curation of the 2000 bp sequences upstream of the start codon of *CoXTHs* revealed 55 putative *cis*-acting regulatory elements across the *CoXTH* promoters (Figure 3A). These elements were categorized into three main groups based on function: response to abiotic and biotic stress, phytohormone response, and growth and development (Figure 3B). Notably, 14 elements, including MYB, MYC, and ARE, were involved in stress response, 13 elements, including ERE, ABRE, and AAGAA-motif, were associated with phytohormone responses, while 28 elements, primarily the light-responsive elements such as G-box, Box4, and GATA-motif, were linked to plant growth and development.

### 2.5. Expression of CoXTHs Under Drought Stress

**Figure 4:**
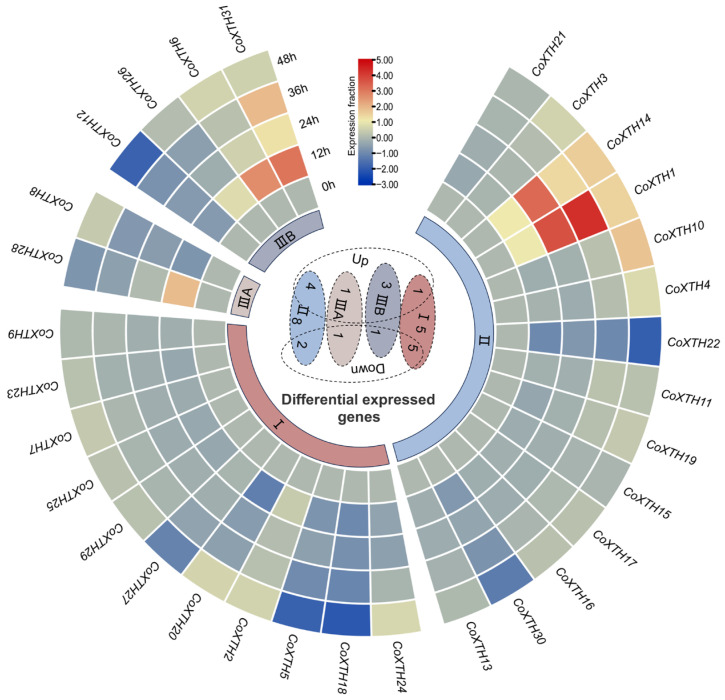
Circos plot of *CoXTH* family genes in one-year-old drought-treated *C. oleifera* seedlings. Samples were collected at 0, 12, 24, 36, and 48 h after treatment. *CoGAPDH* was used as the internal control for determining the expression of the 31 *CoXTH* genes. The middle Venn diagram shows the number of up and down-regulated genes in different subgroups. The outside loop denotes the expression levels of different subgroup classifications. The data are expressed as means ± SD (*n* = 3). Different letters denote significant differences at *p* < 0.05.

Figure 4 and Figure S3 elucidate the response mechanism of the *CoXTH* gene family under simulated drought stress induced by 20% PEG6000 treatment. More than 58% of the *CoXTH* genes responded to drought stress with varying expression levels. *CoXTH1*, *CoXTH6*, *CoXTH14*, *CoXTH28*, and *CoXTH31* were significantly upregulated, with over 2-fold expression increases. In contrast, *CoXTH5*, *CoXTH8*, *CoXTH12*, *CoXTH18*, *CoXTH20*, *CoXTH22*, and *CoXTH30* were significantly downregulated, with over 2-fold expression reductions.

### 2.6. Drought Resistance of CoXTHs in Yeast

**Figure 5:**
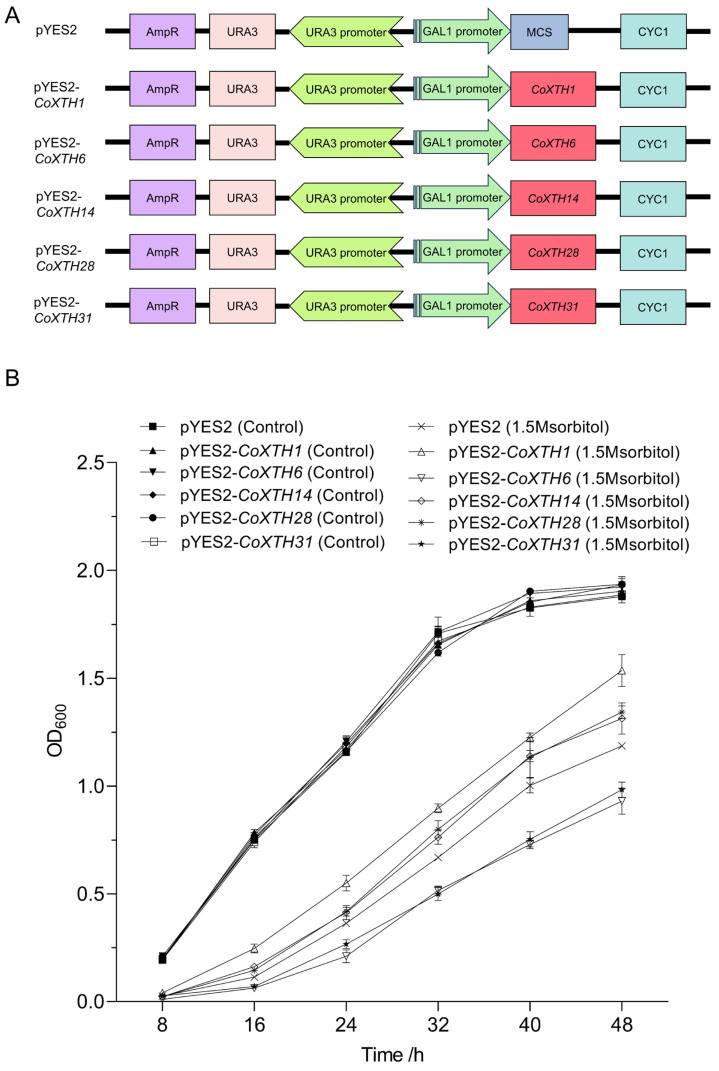
Drought resistance of *CoXTH1*, *CoXTH6*, *CoXTH14*, *CoXTH28*, and *CoXTH31* in yeast. (**A**) Schematic diagrams of *CoXTH1*, *CoXTH6*, *CoXTH14*, *CoXTH28*, and *CoXTH31* expressed vectors. pYES2, and empty vectors (*EV*). pYES2–*CoXTH1*, pYES2–*CoXTH6*, pYES2–*CoXTH14*, pYES2–*CoXTH28*, and pYES2–*CoXTH31* are the recombinant pYES2 vectors that contained *CoXTH1*, *CoXTH6*, *CoXTH14*, *CoXTH28*, and *CoXTH31*, respectively; (**B**) The heterologously expressed yeast strains pYES2, pYES2-*CoXTH1*, pYES2-*CoXTH6*, pYES2-*CoXTH14*, pYES2-*CoXTH28*, and pYES2-*CoXTH31* were subjected to SG/-Ura liquid medium containing 1.5 M sorbitol, measuring the OD600 every 8 h until 48 h. The data are expressed as mean ± SD (*n* = 3).

*CoXTH1*, *CoXTH6*, *CoXTH14*, *CoXTH28*, and *CoXTH31* exhibited significant upregulation under drought stress and the crucial candidate genes associated with drought tolerance in *C. oleifera*. Yeast strains expressing *CoXTH1*, *CoXTH14*, and *CoXTH28* exhibited significantly higher survival rates than the *EV*. In contrast, yeast strains expressing *CoXTH6* and *CoXTH31* demonstrated significantly lower survival rates than the control group (Figure 5 and Figure S4). These findings suggested that *CoXTH1*, *CoXTH14*, and *CoXTH28* positively contribute to plant resistance against drought stress, while *CoXTH6* and *CoXTH31* potentially act as negative regulators of drought tolerance.

### 2.7. Overexpressing CoXTH1 in A. thaliana Enhances Its Drought Tolerance

**Figure 6:**
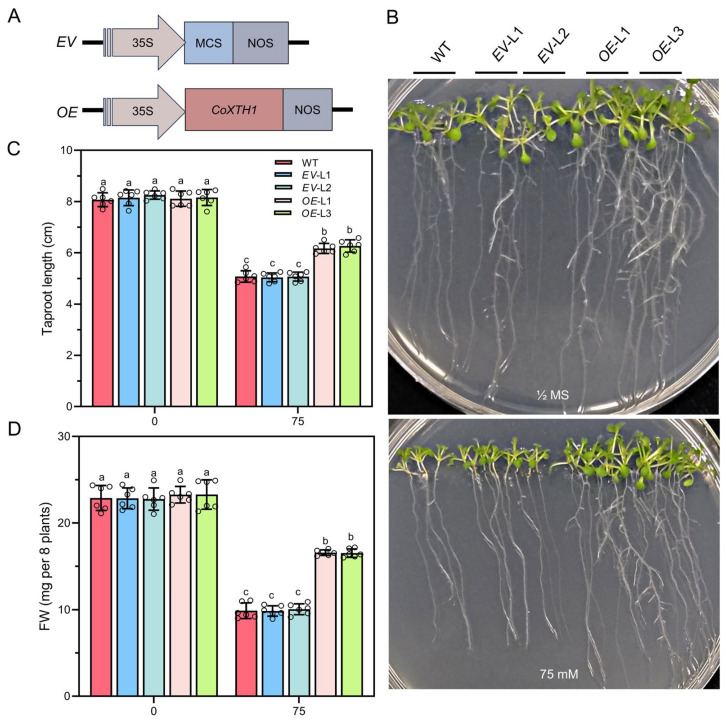
Heterologous *CoXTH1* overexpression enhances drought resistance in *A. thaliana*. (**A**) Schematic diagrams of the *CoXTH1* overexpressed vector. *EV*, empty vector. *OE*, *CoXTH1* overexpressed vector; (**B**) Analysis of drought resistance in *A. thaliana* WT, *EV*, and *OE* seedlings; (**C**,**D**) Root length and FW of the plants described in (**B**). WT, *EV*, and *OE* seedlings were grown on 1/2 MS medium containing 0 and 75 mM mannitol for 10 days. The data are expressed as means ± SD (*n* = 6). Individual data points are represented by circle symbols. Different letters indicate significant differences at *p* < 0.05.

Expressing *CoXTH1* in yeast significantly enhanced its drought tolerance. The cloned *CoXTH1* sequence between *CoXTH1* and *PtrXTH38* exhibited high homology (72.30%, Figure S5), validating its function. The *CoXTH1* coding region was inserted into the pBI121 vector (Figure 6A) to generate transgenic *Arabidopsis* plants overexpressing *CoXTH1* using *Agrobacterium*-mediated transformation. Notably, the expression levels of *CoXTH1* in the nine transgenic *A. thaliana* lines were significantly high (Figure S6). Two high-expression lines, *CoXTH1*-overexpressing (*OE*)*-L1* and *OE-L3*, were subsequently used to establishT3 homozygous lines through kanamycin resistance screening and PCR identification. Figure 6B–D show phenotypic analysis of Wild-type (WT), *EV*, and *OE* lines under drought stress. Of note, there were no significant differences in root length and fresh weight (FW) under normal conditions. However, *OE* lines exhibited significantly greater root length and fresh weight than WT and *EV* under drought stress induced by 75 mM mannitol in 1/2 Murashige and Skoog (MS) medium, confirming that *CoXTH1* overexpression enhances drought tolerance in *A. thaliana*.

### 2.8. Overexpressing CoXTH1 Enhances the ROS Scavenging Ability of A. thaliana

**Figure 7:**
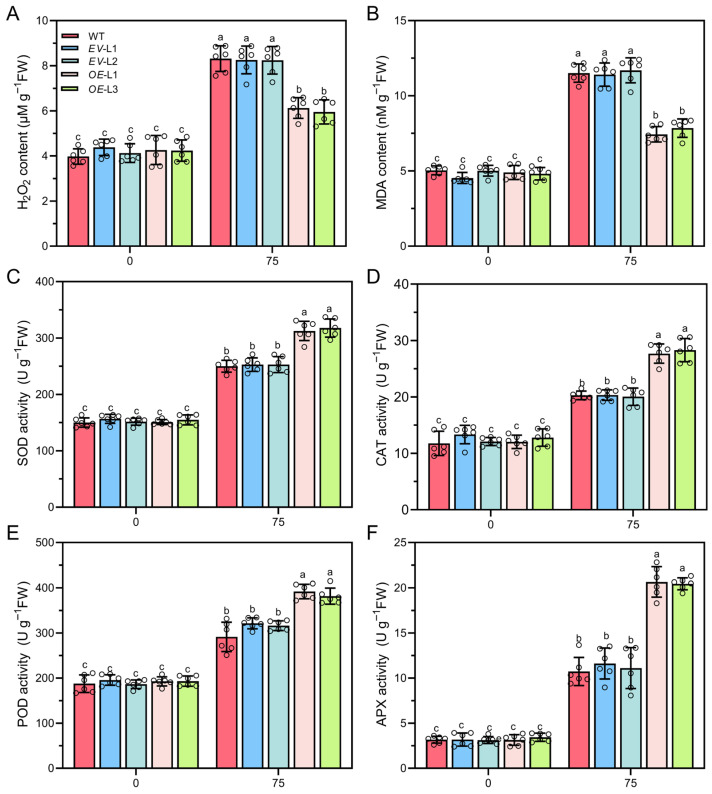
Heterologous *CoXTH1* overexpression reduces reactive ROS, thereby conferring drought resistance in *A. thaliana*. (**A**,**B**) The contents of H_2_O_2_ and MDA in *A. thaliana* plants; (**C**–**F**) The activities of SOD, CAT, POD, and APX in *A. thaliana* plants. WT, *EV*, and *OE* seedlings were grown on 1/2 MS medium containing 0 and 75 mM mannitol for 10 days, before measuring the ROS scavenging ability. The data are expressed as means ± SD (*n* = 6). Individual data points are represented by circle symbols. Different letters indicate significant differences at *p* < 0.05.

Hydrogen peroxide (H_2_O_2_) and malondialdehyde (MDA) are widely recognized biomarkers for evaluating cell membrane damage [11]. In this study, the levels of H_2_O_2_ and MDA were quantified in WT, *EV*, and *OE A. thaliana* plants (Figure 7). Notably, there were no significant differences in H_2_O_2_ and MDA concentrations between WT and *CoXTH1* transgenic plants under normal conditions. However, drought stress significantly increased H_2_O_2_ and MDA levels across all plants, with concentrations rising in response to the stress intensity. Notably, overexpression of *CoXTH1* significantly suppressed the accumulation of H_2_O_2_ and MDA under drought stress. Moreover, there were no notable differences detected in the activities of superoxide dismutase (SOD, EC 1.15.1.1), catalase (CAT, EC 1.11.1.6), ascorbate peroxidase (APX, EC1.1.11.1), and peroxidase (POD, EC 1.11.1.7). among the different plant lines under normal conditions. However, drought stress significantly elevated the activities of these antioxidant enzymes in the overexpression *CoXTH1* transgenic plants compared to the WT and *EV* plants.

### 2.9. Overexpressing CoXTH1 Improves XTH Activity to Increase the Hemicellulose Content of A. thaliana

**Figure 8:**
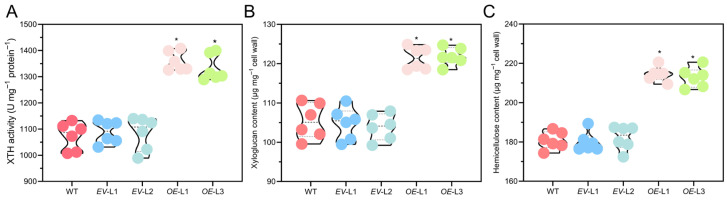
Overexpressing *CoXTH1* enhances xyloglucan synthesis in *A. thaliana*. (**A**) XTH activity; (**B**,**C**) The contents of hemicellulose and xyloglucan in *A. thaliana*, respectively. WT, *EV*, and *CoXTH1 OE* seedlings were grown on 1/2 MS medium containing 0 and 75 mM mannitol for 10 days. The data are expressed as means ± SD (*n* = 6). Individual data points are represented by circle symbols. * means *p* < 0.05.

The XTH enzyme can incorporate newly synthesized xyloglucan into the primary cell wall through its transferase activity [11]. In this study, XTH enzyme activity was significantly higher in *OE A. thaliana* than in WT and *EV* under normal growth and drought stress (Figure 8A). Notably, overexpressing *CoXTH1* in *A. thaliana* substantially increased the xyloglucan contents (Figure 8B). Moreover, overexpressing *CoXTH1* significantly increased the hemicellulose contents (Figure 8C) and moderately elevated cellulose levels, but had no significant effect on pectin and lignin contents (Figure S7). These results indicate that *CoXTH1* could modulate cell wall architecture by enhancing XTH enzymatic activity.

## 3. Discussion

The XTH enzyme is a cell wall remodeling enzyme that plays a crucial role in cell wall structure [26]. Herein, 33 *CoXTH* genes were identified in the *C. oleifera* genome (Figure 1), which were slightly fewer than those reported in dicots, such as *A. thaliana* (33) [18] and *P. trichocarpa* (41) [19]. This difference is attributed to gene loss, fusion during evolution, or incomplete genome assembly that limits complete gene detection, as reported in other plant gene family studies [27]. In a previous study [17], 31 *CoXTH* genes were grouped into four subfamilies. Groups I and II contain the most members (Figure 1B), suggesting functional diversity and evolutionary conservation among XTH subgroups. Previous studies postulate that motif, a vital catalytic site for XET and XEH activities, is highly conserved among all identified XTH proteins [16]. In this study, all CoXTH proteins contained the same conserved domain (Figure S1). However, the DEIDFEFLG motif contained some amino acid substitutions across the CoXTH family members. For instance, the fifth phenylalanine (F) in CoXTH7 was replaced by isoleucine (I). However, earlier research indicates that such changes do not significantly affect enzyme activity or function [28]. Additionally, N-glycosylation sites near the catalytic motif in Group IIIA of CoXTH proteins of *C. oleifera* contained amino acid residue variations (Figure S2). Similar variations exist in Group IIIA of XTH proteins of *A. thaliana* and *O. sativa* [25], corroborating the idea that the *XTH* gene family is evolutionarily conserved across species.

Tandem and segmental duplication events are primary driving forces in the expansion and evolution of gene families [29]. The *XTH* gene family in plants has also undergone such events [30]. Herein, *C. oleifera* had four pairs of duplicated *CoXTH* family genes arising from segmental duplication events (Figure 2A), suggesting that tandem duplication was potentially not the primary mechanism regulating the expansion of the *CoXTH* gene family in *C. oleifera*. Similarly, segmental duplication events are the dominant mode of *XTH* gene family expansion in *Z. mays* [22] and *Ipomoea batatas* [31]. The *XTH* gene pairs resulting from segmental duplication in *C. oleifera* exhibited high protein sequence similarity among them. Moreover, their gene structures and conserved protein motifs exhibited considerable consistency (Figure S1). However, these gene pairs showed significantly different expression patterns under drought stress despite the high level of sequence similarity (Figure 4 and Figure S3), suggesting that functional divergence may have occurred during *C. oleifera* adaptation to environmental changes. Comparative collinearity analysis of *XTH* genes across species further revealed more syntenic gene pairs between *C. oleifera* and dicotyledonous species, including *A. thaliana* and *P. trichocarpa* than with monocotyledonous species, such as *O. sativa* and *Z. mays* (Figure 2B). This pattern potentially reflects the distinct evolutionary trajectories and expansion dynamics of *XTH* genes in monocots and dicots, indicating independent evolution following the divergence of these plant lineages. Similar observations have been reported in other species, including the *ZmXTH* gene family in maize [22] and the *CAX* gene family in *P. trichocarpa* [32].

Promoters, located upstream of the gene coding sequences, act as vital regulatory switches controlling gene transcription and expression. They contain various *cis*-acting regulatory elements crucial for signal transduction and responses to environmental stimuli [16]. Transcription factors (TFs), a class of regulatory proteins, regulate target gene expression by specifically binding to these *cis*-regulatory elements [33]. In this study, the promoter regions of the *CoXTH* gene family, including binding sites for MYC and MYB transcription factors, contained several TF-related *cis*-regulatory motifs (Figure 3 and Table S3). These elements allow rapid activation of the *CoXTH* genes during different developmental stages and under stress conditions, enhancing plant adaptability. Additionally, hormone signaling molecules regulate gene expression by interacting with specific *cis*-acting elements in promoters [34]. For example, methyl jasmonate (MeJA) affects the promoter activity of *BnXTH1* in *Boehmeria nivea*, thereby improving its cadmium tolerance [11]. Similarly, the *CoXTH* gene family promoters studied herein contained multiple hormone-responsive *cis*-acting elements, including the CGTCA-motif, AAGAA-motif, and F-box elements linked to ABA, ET, GA, and MeJA signaling pathways (Figure 3 and Table S3). Therefore, the *CoXTH* gene family potentially regulates plant growth and stress responses through hormonal signal perception and integration. Notably, current knowledge about *CoXTH* promoters primarily relies on computational predictions and thus requires experimental validation.

The cell wall, which is the outermost structural layer of plant cells, provides mechanical support and plays a crucial role in plants’ response to environmental stresses. Water stress can alter the cell wall composition, influencing its extensibility [12]. XTH-mediated cell wall modifications contribute to maintaining the structural integrity of plant cells [35]. This study revealed that *CoXTH1* is closely associated with plant drought resistance. Its overexpression in *A. thaliana* significantly enhanced tolerance to drought stress (Figure 6B–D). ROS accumulate excessively in plants, leading to cellular damage and impaired growth and development under stress conditions [36]. Overexpressing *CoXTH1* in *A. thaliana* significantly increased antioxidant enzyme activity and effectively reduced ROS accumulation under drought stress (Figure 7). These findings suggest that *CoXTH1* potentially enhances cell structural stability by regulating cell wall modifications, thereby improving the capacity of the plant to scavenge ROS and increase its adaptability to drought stress.

Previous studies postulate that Group II proteins exhibit XET activity [37]. CoXTH1 is categorized within Group II (Figure 1B), suggesting that it also possesses XET activity, which enables the integration of newly secreted xyloglucan oligosaccharides into pre-existing xyloglucan chains, thereby increasing the xyloglucan content within the cell wall. This mechanism aligns with the findings of this study, which demonstrated enhanced XTH activity and increased xyloglucan levels following heterologous overexpression of *CoXTH1* in *A. thaliana* (Figure 8A,B). In dicotyledonous plants, xyloglucan is the major component of hemicellulose. Changes in its content directly affect total hemicellulose levels [11]. Herein, overexpressing *CoXTH1* significantly increased the hemicellulose content in *A. thaliana* (Figure 8C), possibly because of a corresponding rise in the xyloglucan content. In the plant cell wall, xyloglucan exhibits a strong binding affinity for cellulose, facilitating the crosslinking of adjacent cellulose microfibrils [38]. Previous studies postulate that variations in xyloglucan content affect cellulose biosynthesis [39]. In this study, overexpressing *CoXTH1* increased the cellulose content in *A. thaliana* (Figure S7A), suggesting that heterologous *CoXTH1* expression potentially promotes cellulose synthesis. Similar findings have been reported in *A. thaliana* expressing *AtXTH21* [40] and *AtXTH30* [41], which participate in cellulose biosynthesis. However, the exact mechanism by which *CoXTH1* enhances cellulose synthesis through heterologous expression remains unclear.

## 4. Materials and Methods

### 4.1. Identification of the CoXTH Gene Family

Potential *CoXTH* genes were identified within the *C. oleifera* genome dataset (Genbank accession number: GCA_025200525.1) [42] using the Hidden Markov Model (HMM) constructed from two conserved domains, PF00722 and PF06955, of the *XTH* gene family [23]. Redundant sequences were removed through manual curation to obtain a preliminary set of candidate genes. *A. thaliana* AtXTH protein sequences were obtained from the National Center for Biotechnology Information (NCBI) Protein database (http://www.ncbi.nlm.nih.gov/protein/, accessed on 18 May 2024). The Simple HMM Search plugin in TBtools v2.102 [43] was utilized to conduct homologous alignment analysis against the *C. oleifera* genome, further refining the list of candidate genes. The results from both screening approaches were finally combined and subjected to manual curation to eliminate duplicates, thus defining the complete *CoXTH* gene family.

The ExPASy-ProtParam software (https://web.expasy.org/protparam/, accessed on 4 June 2024) [44] was used to analyze the physicochemical properties of the CoXTH family proteins, while the subcellular localization of members within this gene family was predicted using the Plant-mPLoc software (http://www.csbio.sjtu.edu.cn/bioinf/plant-multi/, accessed on 4 June 2024) [45]. MG2C v2.1 (http://mg2c.iask.in/mg2c_v2.1/, accessed on 13 June 2024) [46] was used to visually analyze the chromosomal distribution of the *CoXTH* gene family.

### 4.2. Phylogenetic Relationship, Gene Structure, and Conserved Motif Analysis of the CoXTH Gene Family

MEGA 7.0 software [47] was employed to align the CoXTH and AtXTH protein sequences, followed by the construction of a phylogenetic tree using NJ method with 1000 bootstrap replicates. The genomic and CDS of the *CoXTH* gene family were extracted from the *C. oleifera* genome, and the gene structure of the *CoXTH* gene family was subsequently visualized using Gene Structure Display Server (GSDS 2.0 http://gsds.gao-lab.org/, accessed on 3 July 2024) [48]. The Multiple Em for Motif Elicitation (MEME) online tool (https://meme-suite.org/meme/tools/meme, accessed on 3 July 2024) [49] was then applied to analyze the conserved motifs of the CoXTH protein sequences. Ten conserved motifs were identified, and their results were graphically displayed using the TBtools software [43].

### 4.3. Gene Duplication and Collinearity Analysis of the CoXTH Gene Family

The genomic data of *A. thaliana*, *O. sativa*, *C. sinensis*, and *P. trichocarpa* were retrieved from the NCBI GenBank database (https://www.ncbi.nlm.nih.gov/datasets/genome, accessed on 9 July 2024). The One Step MCScanX plugin in the TBtools software was subsequently applied to identify the *CoXTH* gene family duplication events in the *C. oleifera* genome and examine the collinearity relationships of *XTH* genes between *C. oleifera* and the aforementioned species. TBtools was finally used to present the results of these analyses graphically for visualization.

### 4.4. Cis-Element Analysis of the CoXTH Gene Family Promoter

The 2000bp DNA sequence upstream of the *CoXTH* gene start codon was retrieved from the *C. oleifera* genome and used as the candidate promoter region for further analysis. The PlantCare tool (https://bioinformatics.psb.ugent.be/webtools/plantcare/html/, accessed on 14 July 2024) [50] was then used to predict potential *cis*-acting regulatory elements within this sequence. TBtools was finally employed to graphically visualize the predicted results.

### 4.5. Expression Analysis of the CoXTH Gene Family Under Drought Stress

One-year-old *C. oleifera* cuttings were pre-cultured in 1/2 Hoagland’s nutrient solution for 14 days under a 16 h light/8 h dark photoperiod, 26 °C, 70% relative humidity, and a light intensity of 20,000 lux. Drought stress was subsequently induced by supplementing the culture medium with 20% PEG6000 [51]. Root, stem, and leaf tissues were collected in liquid nitrogen at 0, 12, 24, 36, and 48 h after treatment initiation for further analysis. Total RNA was extracted using the Plant RNA Extraction Kit (TIANGEN, Beijing, China), following the manufacturer’s instructions. A260/A280 ratios of all the RNA samples were in the approximate range of 1.8–2.0 based on a Nanodrop spectrophotometer. The RNA samples were then reverse-transcribed into cDNA using the TaKaRa Bio Reverse Transcription Kit (Tokyo, Japan), followed by RT-qPCR amplification of the target genes on an ABI QuantStudio™ 6 Flex qPCR platform (Applied Biosystems, Waltham, MA, USA). Gene-specific primers were synthesized by Tsingke Biotech Co., Ltd. (Beijing, China). Table S4 lists the target genes and their sequences. The TB Green^®^ Premix Ex Taq™ II FAST qPCR Kit (TaKaRa Bio, Tokyo, Japan) was used for RT-qPCR. The RT-qPCR program was as follows: reverse transcription step at 25 °C for 10 min, followed by initial denaturation at 95 °C for 30 s, and 40 cycles of denaturation and primer annealing at 95 °C for 10 s and 60 °C for 10 s, respectively. Each RT-qPCR reaction was prepared as a 25 µL volume consisting of 12.5 µL of 2× reaction mix, 1.0 µL of each primer, and 2 µL of cDNA template. *CoGAPDH* was used as the internal control [51]. Each sample was amplified in triplicate, followed by a calculation of the relative expression levels of the target genes using the 2^−ΔΔCt^ method.

### 4.6. Heterologous Expression and Drought Resistance Assays of CoXTHs in Yeast

*CoXTH1*, *CoXTH6*, *CoXTH14*, *CoXTH28*, and *CoXTH31* genes exhibited significant positive responses to drought stress and were identified through RT-qPCR analysis. The CDS of these genes were cloned into the pYES2 vector and subsequently introduced into the yeast strain INVSc1, resulting in transgenic yeast strains harboring pYES2-*CoXTH1*, pYES2-*CoXTH6*, pYES2-*CoXTH14*, pYES2-*CoXTH28*, and pYES2-*CoXTH31* constructs. The transformed yeast cells were subjected to 3.5 M sorbitol stress for 24 h and then spotted onto SG/-Ura selective medium plates to assess their growth performance, as described by Bi et al. [23]. Freshly prepared transgenic yeast cells were resuspended in liquid culture medium containing 0 and 1.5 M sorbitol, respectively, to compare the osmotic stress responses of transgenic yeast strains. The cultures were incubated at 30 °C with constant agitation at 250 rpm. Osmotic stress adaptation was evaluated on all replicates by monitoring OD600 values of the yeast transformants under these conditions, following the established protocol described by Jiang et al. [26]. Three replicates were performed.

### 4.7. A. thaliana Transformation and Drought Treatment

The CDS of the *CoXTH1* gene was cloned into the pBI121 vector, which was subsequently introduced into *Agrobacterium tumefaciens* GV3101 to generate the recombinant strain pBI121-*CoXTH1*-GV3101. The recombinant strain was subsequently used for the genetic transformation of *A. thaliana* using the floral dip method described by Clough and Bent [52]. Transgenic *A. thaliana* lines carrying *EV* and *OE* were obtained and confirmed through kanamycin resistance screening and PCR analysis. Two transgenic lines (L1 and L3) with high expression levels were selected and self-fertilized to produce T_3_ homozygous lines. Seeds from these lines were collected for use in subsequent experiments.

Surface-sterilized WT, *EV*, and *OE A. thaliana* seeds were sown on 1/2 MS medium and let to grow for 3 days, after which the seedlings were transferred to 1/2 MS medium containing either 0 mM or 75 mM mannitol and cultured vertically to test for drought tolerance. Tolerance indices, including root length and FW, were recorded after 10 days. Plant samples were also collected for physiological and biochemical analyses, including enzymatic activity and xyloglucan content.

### 4.8. Determination of H_2_O_2_, MDA, and Antioxidant Enzyme Activity

The samples were ground in liquid nitrogen before measuring H_2_O_2_ and MDA levels, as well as SOD, CAT, APX, and POD activities. The MDA content was quantified using the thiobarbituric acid method (TBA method), following the procedure described by Li and Chow [53]. The H_2_O_2_ content was determined using the iodometric method as described by Gebicki et al. [54]. The enzymatic activities of SOD, CAT, APX, and POD were assessed using their respective assay kits following the requisite manufacturer’s instructions (Solarbio, Beijing, China). Each sample was analyzed in sextuplicate.

### 4.9. Measurement of Cell Wall Fraction and Xyloglucan Content

The cell wall components and xyloglucan content were analyzed using the method described by Ma et al. [11]. Briefly, samples were ground in liquid nitrogen and washed with cold organic solvents, including 75% ethanol, acetone, methanol/chloroform (1:1, *v*/*v*), and methanol, to isolate the purified cell wall material. Sequential extractions of the cell walls were then performed using boiling water and sodium hydroxide solution to obtain distinct fractions of pectin, hemicellulose, cellulose, and lignin. Cellulose and hemicellulose contents were quantified using the phenol-sulfuric acid method, while pectin and lignin contents were measured using the carbazole colorimetric assay and the acetyl bromide method, respectively. The iodine staining method was finally used to assess the xyloglucan content within the hemicellulose extract [13]. Each sample was analyzed in sextuplicate.

### 4.10. XTH Activities Assay

*A. thaliana* samples were ground in liquid nitrogen, mixed with an enzyme extraction buffer, and then centrifuged. The resulting supernatant was collected as the crude enzyme extract. The protein content in the crude enzyme solution was quantified using the Coomassie Brilliant Blue G-250 binding method [55]. XTH activity was determined using an ELISA kit (MEIMIAN, Shanghai, China), following the manufacturer’s instructions. The measured activity was subsequently normalized based on the protein content per unit mass. Each sample was analyzed in sextuplicate.

### 4.11. Statistical Analysis

All data were subjected to a one-way analysis of variance (ANOVA), followed by Tukey’s multiple comparison test for post hoc comparisons with a significance level of *p* < 0.05. The correlation was analyzed by the Data Processing System (DPS) software (version 9.01, China). The results are expressed as means ± standard deviation (SD).

## 5. Conclusions

Herein, the *CoXTH* gene family in *C. oleifera* was systematically identified and analyzed. Notably, 31 *CoXTH* genes unevenly distributed across 14 chromosomes were identified and categorized into four subfamilies. 18 *CoXTH* genes responded to drought stress. *CoXTH1*, *CoXTH6*, *CoXTH14*, *CoXTH28*, and *CoXTH31* were significantly upregulated, while *CoXTH5*, *CoXTH8*, *CoXTH12*, *CoXTH18*, *CoXTH20*, *CoXTH22*, and *CoXTH30* were significantly downregulated. Heterologous expression of *CoXTH1* in yeast significantly improved yeast survival under high sorbitol concentrations. Heterologous expression of *CoXTH1* in *A. thaliana* enhanced XTH enzyme activity by over 26%, leading to high xyloglucan content and subsequent accumulation of hemicellulose and cellulose in the cell wall. This structural modification improved cell wall stability under drought stress, ultimately enhancing plant drought tolerance. The findings of this study highlight the importance of *CoXTH1* in drought stress adaptation, and reveal candidate genes for future biotechnological strategies to improve drought tolerance in woody crops.

## Figures and Tables

**Table 1:** Molecular characterization of *CoXTH* genes.

Name	Genome Location	PI	Mw (kDa)	Peptide Residue (aa)	Gravy	Aliphatic Index	CDS Length (bp)	Predicted Subcellular Localization
*CoXTH1*	Chr1:148173832-148175859	6.81	32.24	287	−0.322	73.41	864	Cell wall or Cytoplasm
*CoXTH2*	Chr1:184263085-184266113	7.04	32.90	287	−0.309	74.70	864	Cell wall
*CoXTH3*	Chr2:14125984-14127001	6.59	32.01	283	−0.226	74.81	852	Cell wall or Cytoplasm
*CoXTH4*	Chr2:15002727-15005660	4.73	33.43	297	−0.374	75.22	894	Cell wall
*CoXTH5*	Chr2:185621304-185624087	6.44	33.39	293	−0.363	68.91	882	Cell wall
*CoXTH6*	Chr3:144786121-144788984	8.97	39.27	341	−0.549	67.18	1026	Cell wall
*CoXTH7*	Chr3:58816285-58819353	6.04	31.60	276	−0.471	79.29	831	Cell wall
*CoXTH8*	Chr4:29353162-29355745	9.31	33.75	294	−0.339	63.06	885	Cell wall
*CoXTH9*	Chr4:43136898-43148390	9.11	36.83	319	−0.461	81.25	960	Cell wall
*CoXTH10*	Chr4:117068255-117071186	4.81	28.63	256	−0.198	80.43	771	Cell wall
*CoXTH11*	Chr5:127002401-127004274	8.45	32.49	284	−0.367	64.51	855	Cell wall or Cytoplasm
*CoXTH12*	Chr6:11102489-11105784	6.13	35.19	311	−0.188	77.72	936	Cell wall
*CoXTH13*	Chr6:54452832-54463757	8.21	33.53	291	−0.449	60.72	876	Cell wall
*CoXTH14*	Chr7:91156960-91242883	5.36	32.07	288	−0.341	69.10	867	Cell wall or Cytoplasm
*CoXTH15*	Chr7:112011888-112012997	7.58	32.53	289	−0.252	72.56	870	Cell wall
*CoXTH16*	Chr7:112063339-112064355	9.25	30.10	261	−0.526	63.87	786	Cell wall
*CoXTH17*	Chr7:112080286-112081540	8.58	38.44	336	−0.372	68.18	1011	Cell wall or Cytoplasm
*CoXTH18*	Chr7:136193032-136194628	5.99	33.79	297	−0.308	67.58	894	Cell wall
*CoXTH19*	Chr8:76121722-76124240	8.82	33.21	293	−0.348	64.91	882	Cell wall or Cytoplasm
*CoXTH20*	Chr8:117607450-117609597	5.45	39.09	337	−0.557	58.46	1014	Cell wall
*CoXTH21*	Chr8:135166938-135167942	8.80	38.18	334	−0.500	58.71	1005	Cell wall or Cytoplasm
*CoXTH22*	Chr9:136941105-136943159	9.25	32.08	281	−0.326	71.78	846	Cell wall or Cytoplasm
*CoXTH23*	Chr9:145532451-145534317	8.72	33.11	286	−0.490	67.13	861	Cell wall
*CoXTH24*	Chr10:891170-894544	7.65	34.12	295	−0.465	65.42	888	Cell wall or Cytoplasm
*CoXTH25*	Chr11:32609393-32611893	5.41	32.29	282	−0.661	59.82	849	Cell wall
*CoXTH26*	Chr12:29226967-29229771	6.17	36.39	320	−0.495	71.84	963	Cell wall
*CoXTH27*	Chr13:32352163-32354989	8.23	35.13	302	−0.374	72.95	909	Cell wall
*CoXTH28*	Chr13:81713234-81715882	5.33	32.84	291	−0.453	61.65	876	Cell wall
*CoXTH29*	Chr13:123918650-123921005	5.88	34.71	298	−0.412	74.90	897	Cell wall
*CoXTH30*	Chr14:43790930-43793214	6.20	33.50	289	−0.398	63.11	870	Cell wall
*CoXTH31*	Chr14:108417298-108423064	9.11	40.14	352	−0.414	68.47	1059	Cell wall

## Data Availability

The original contributions presented in this study are included in the article/Supplementary Material. The *CoXTH* gene family and protein sequences were deposited in GenBank (PX547902−PX547932). Further inquiries can be directed to the corresponding authors.

## Supplementary Materials

The following supporting information can be downloaded at: https://www.mdpi.com/article/10.3390/plants14233605/s1, Figure S1: Gene structure and conserved motifs of CoXTH family genes; Figure S2: The active catalytic regions of CoXTH protein family; Figure S3: RT- qPCR analysis of the CoXTH genes under drought stress; Figure S4: Comparison of the growths of osmotolerance yeast cells; Figure S5: Sequence analysis of CoXTH1; Figure S6: RT-qPCR analysis of the CoXTH1 gene in different; Figure S7: The content of cellulose (A), pectin (B), and lignin(C) in A. thaliana; Table S1: Sequence and SeqLogo of the Motif 1-10; Table S2: Collinear of XTH genes among different plants; Table S3: Cis-acting elements analyses of the CoXTHs gene promoter; Table S4: Primers used in the study.
