# Supplementary material for: Genome-Wide Identification and Characterization of the Xyloglucan Endotransglucosylase/Hydrolase (XTH) Gene Family in Camellia oleifera and the Function of CoXTH1 During Drought Stress
Source: Plants (Basel). 2025 Nov 26;14(23):3605. doi: 10.3390/plants14233605 (PMC12694087; doi:10.3390/plants14233605)
Supplement: Supplementary file 1 [file plants-14-03605-s001.zip › plants-3960151-supplementary.pdf]

Supplementary information

Table S1. Sequence and SeqLogo of the Motif 1-10

Motif ID	SeqLogo	Amino acid sequence encode
Motif_1	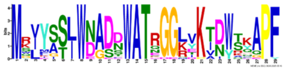	MRVYSSJWBADBWATRGGLVKTDWTKAPF
Motif_2	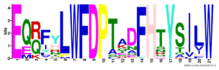	EQRFYLWFDPTADFHTYSILW
Motif_3	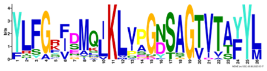	YLFGRFDMQJKLVPGBSAGTVTAYYL
Motif_4	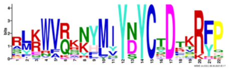	RLKWVRKNYMIYBYCTDTKRFP
Motif_5	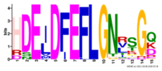	HDEJDFEFLGNRSGQ
Motif_6	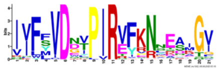	IVFFVDNVPIRVFKNNEAIGV
Motif_7	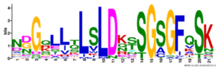	NDGQLLTLSLDKSSGSGFQSK
Motif_8	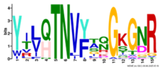	YILQTNVYAQGKGNR
Motif_9	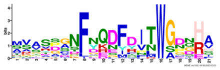	MVASSGNFNQDFDITWGBNHA
Motif_10	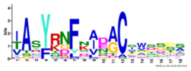	IASYRNFKADACSWSSPS

Table S2. Collinear of *XTH* genes among different plants

C. oleifera-A. thaliana	*C. oleifera-P. trichocarpa*	*C. oleifera-O. sativa*	*C. oleifera-Z. mays*
CoXTH2-AtXTH7	*CoXTH15-PtXTH12*	*CoXTH13-OsXTH13*	*CoXTH13-ZmXTH12*
CoXTH2-AtXTH6	*CoXTH15-PtXTH28*	*CoXTH20-OsXTH1*	*CoXTH20-ZmXTH18*
CoXTH5-AtXTH7	*CoXTH18-PtXTH30*	*CoXTH26-OsXTH28*	*CoXTH30-ZmXTH15*
CoXTH3-AtXTH25	*CoXTH18-PtXTH35*	*CoXTH30-OsXTH13*	
CoXTH5-AtXTH6	*CoXTH20-PtXTH25*		
CoXTH8-AtXTH32	*CoXTH20-PtXTH16*		
CoXTH12-AtXTH33	*CoXTH22-PtXTH21*		
CoXTH13-AtXTH26	*CoXTH22-PtXTH14*		
CoXTH25-AtXTH3	*CoXTH24-PtXTH27*		
CoXTH26-AtXTH28	*CoXTH24-PtXTH34*		
CoXTH26-AtXTH27	*CoXTH25-PtXTH15*		
CoXTH28-AtXTH32	*CoXTH26-PtXTH39*		
CoXTH28-AtXTH31	*CoXTH26-PtXTH40*		
	CoXTH28-PtXTH6		
	CoXTH28-PtXTH22		
	CoXTH27-PtXTH29		
	CoXTH28-PtXTH14		
	CoXTH30-PtXTH18		

Table S3. Cis-acting elements analyses of the *CoXTHs* gene promoter

Name	Element name	Site sequence	Postion	function
CoXTH1p	MYB	CAACCA	1992-1997	MYB binding site
CoXTH1p	MYC	CATGTG	517-522	MYC binding site
CoXTH1p	MYC	CATTTG	776-781	MYC binding site
CoXTH1p	MYC	CATGTG	826-831	MYC binding site
CoXTH1p	CGTCA-motif	CGTCA	142-146	Cis-acting regulatory element involved in the MeJA responsiveness
CoXTH1p	STRE	AGGGG	478-482	Osmotic stress response element
CoXTH1p	STRE	AGGGG	492-496	Osmotic stress response element
CoXTH1p	STRE	AGGGG	1530-1534	Osmotic stress response element
CoXTH1p	G-box	CACGTC	140-145	Cis-acting regulatory element involved in light responsiveness
CoXTH1p	G-box	TACGTG	498-503	Cis-acting regulatory element involved in light responsiveness
CoXTH1p	Box 4	ATTAAT	254-259	Part of a conserved DNA module involved in light responsiveness
CoXTH1p	P-box	CCTTTTG	1169-1175	Gibberellin-responsive element
CoXTH1p	ERE	ATTTCATA	121-128	Ethylene response element
CoXTH1p	ERE	ATTTTAAA	1005-1012	Ethylene response element
CoXTH1p	ERE	ATTTTAAA	1192-1199	Ethylene response element
CoXTH1p	I-box	AAGATAAGGCT	1238-1247	Part of a light responsive element
CoXTH1p	MYC	TCTCTTA	935-941	MYC binding site
CoXTH1p	ARE	AAACCA	665-670	Cis-acting regulatory element essential for the anaerobic induction
CoXTH1p	ARE	AAACCA	1218-1223	Cis-acting regulatory element essential for the anaerobic induction
CoXTH1p	WUN-motif	TTATTACAT	921-929	Stress-responsive element
CoXTH1p	WUN-motif	AAATTACT	1075-1082	Stress-responsive element
CoXTH1p	ABRE	ACGTG	499-503	Cis-acting element involved in the abscisic acid responsiveness
CoXTH1p	ABRE3a	TACGTG	498-503	Cis-acting element involved in the abscisic acid responsiveness
CoXTH2p	F-box	CTATTCTCATT	1948-1957	Gibberellin response element

CoXTH2p	TCT-motif	TCTTAC	239-244	Part of a light responsive element
CoXTH2p	TCT-motif	TCTTAC	918-923	Part of a light responsive element
CoXTH2p	TCT-motif	TCTTAC	1055-1060	Part of a light responsive element
CoXTH2p	AAGAA-motif	GAAAGAA	1545-1551	Cis-acting element involved in the abscisic acid responsiveness
CoXTH2p	WRE3	CCACCT	144-149	Wound responsiveness
CoXTH2p	MYC	CATTTG	1688-1693	MYC binding site
CoXTH2p	G-Box	CACGTG	291-296	Cis-acting regulatory element involved in light responsiveness
CoXTH2p	W box	TTGACC	304-309	Stress-responsive element
CoXTH2p	W box	TTGACC	1957-1962	Stress-responsive element
CoXTH2p	Sp1	GGGCGG	1626-1631	Light responsive element
CoXTH2p	LAMP-element	CTTTATCA	945-952	Part of a light responsive element
CoXTH2p	TC-rich repeats	GTTTTCTTAC	235-243	Cis-acting element involved in defense and stress responsiveness
CoXTH2p	ABRE	CACGTG	291-296	Cis-acting element involved in the abscisic acid responsiveness
CoXTH2p	ABRE	ACGTG	292-296	Cis-acting element involved in the abscisic acid responsiveness
CoXTH2p	ABRE	ACGTG	713-717	Cis-acting element involved in the abscisic acid responsiveness
CoXTH2p	Box 4	ATTAAT	88-93	Part of a conserved DNA module involved in light responsiveness
CoXTH2p	Box 4	ATTAAT	594-599	Part of a conserved DNA module involved in light responsiveness
CoXTH2p	Box 4	ATTAAT	911-916	Part of a conserved DNA module involved in light responsiveness
CoXTH2p	G-box	CACGTG	291-296	Cis-acting regulatory element involved in light responsiveness
CoXTH2p	CCAAT-box	CAACGG	809-814	MYBHv1 binding site
CoXTH2p	TGACG-motif	TGACG	111-115	Cis-acting regulatory element involved in the MeJA responsiveness
CoXTH2p	TGACG-motif	TGACG	1638-1642	Cis-acting regulatory element involved in the MeJA responsiveness
CoXTH2p	TCA-element	CCATCTTTTT	659-667	Cis-acting element involved in salicylic acid responsiveness
CoXTH2p	TCA-element	CCATCTTTTT	845-853	Cis-acting element involved in salicylic acid responsiveness
CoXTH2p	CGTCA-motif	CGTCA	1806-1810	Cis-acting regulatory element involved in the MeJA responsiveness
CoXTH2p	MYB	TAACTG	263-268	MYC binding site

CoXTH2p	CCGTCC-box	CCGTCC	655-660	Cis-acting regulatory element related to fruit development
CoXTH2p	ARE	AAACCA	141-146	Cis-acting regulatory element essential for the anaerobic induction
CoXTH2p	GATA-motif	AAGATAAGATT	365-374	Part of a light responsive element
CoXTH2p	AuxRR-core	GGTCCAT	1631-1637	Cis-acting regulatory element involved in auxin responsiveness
CoXTH2p	as-1	TGACG	111-115	Cis-acting element involved in the MeJA responsiveness
CoXTH2p	as-1	TGACG	1638-1642	Cis-acting element involved in the MeJA responsiveness
CoXTH2p	CAT-box	GCCACT	205-210	Cis-acting regulatory element related to meristem expression
CoXTH3p	as-1	TGACG	618-622	Cis-acting element involved in the MeJA responsiveness
CoXTH3p	ERE	ATTTCATA	1393-1400	Ethylene response element
CoXTH3p	MBSI	aaaAaaC(G/C)GTTA	1337-1346.5	MYB binding site involved in flavonoid biosynthetic genes regulation
CoXTH3p	GT1-motif	GGTTAA	14-19	Light responsive element
CoXTH3p	GT1-motif	GGTTAA	982-987	Light responsive element
CoXTH3p	GT1-motif	GGTTAA	1033-1038	Light responsive element
CoXTH3p	ARE	AAACCA	663-668	Cis-acting regulatory element essential for the anaerobic induction
CoXTH3p	ARE	AAACCA	1523-1528	Cis-acting regulatory element essential for the anaerobic induction
CoXTH3p	ARE	AAACCA	1783-1788	Cis-acting regulatory element essential for the anaerobic induction
CoXTH3p	WRE3	CCACCT	361-366	Wound responsiveness
CoXTH3p	WRE3	CCACCT	382-387	Wound responsiveness
CoXTH3p	WRE3	CCACCT	425-430	Wound responsiveness
CoXTH3p	WRE3	CCACCT	1621-1626	Wound responsiveness
CoXTH3p	MYB	TAACCA	379-384	MYB Binding Site
CoXTH3p	MYB	TAACCA	422-427	MYB Binding Site
CoXTH3p	chs-CMA1a	TTACTTAA	1797-1804	Part of a light responsive element
CoXTH3p	CAT-box	GCCACT	115-120	Cis-acting regulatory element related to meristem expression
CoXTH3p	CAT-box	GCCACT	1641-1646	Cis-acting regulatory element related to meristem expression
CoXTH3p	MYB	TAACCA	379-384	MYB Binding Site

CoXTH3p	MYB	TAACCA	422-427	MYB Binding Site
CoXTH3p	MYB	CAACCA	1969-1974	MYB Binding Site
CoXTH3p	MYC	TCTCTTA	857-863	MYC binding site
CoXTH3p	MYC	CAATTG	277-282	MYC binding site
CoXTH3p	MYC	CATTTG	300-305	MYC binding site
CoXTH3p	AAGAA-motif	GAAAGAA	174-180	Cis-acting element involved in the abscisic acid responsiveness
CoXTH3p	AAGAA-motif	GAAAGAA	178-184	Cis-acting element involved in the abscisic acid responsiveness
CoXTH3p	AAGAA-motif	GAAAGAA	806-812	Cis-acting element involved in the abscisic acid responsiveness
CoXTH3p	GATA-motif	AAGGATAAGG	143-151	Part of a light responsive element
CoXTH3p	TGACG-motif	TGACG	618-622	Cis-acting regulatory element involved in the MeJA responsiveness
CoXTH3p	TCA-element	CCATCTTTTT	361-369	Cis-acting element involved in salicylic acid responsiveness
CoXTH3p	TCA-element	CCATCTTTTT	1419-1427	Cis-acting element involved in salicylic acid responsiveness
CoXTH3p	ABRE	ACGTG	1768-1772	Cis-acting element involved in the abscisic acid responsiveness
CoXTH3p	ABRE	ACGTG	1881-1885	Cis-acting element involved in the abscisic acid responsiveness
CoXTH3p	Box 4	ATTAAT	929-934	Part of a conserved DNA module involved in light responsiveness
CoXTH3p	WUN-motif	AAATTTCTT	1458-1466	Stress-responsive element
CoXTH4p	CGTCA-motif	CGTCA	40-44	Cis-acting regulatory element involved in the MeJA responsiveness
CoXTH4p	CGTCA-motif	CGTCA	1087-1091	Cis-acting regulatory element involved in the MeJA responsiveness
CoXTH4p	F-box	CTATTCTCATT	344-353	Gibberellin response element
CoXTH4p	MYC	CATTTG	1011-1016	MYC binding site
CoXTH4p	WRE3	CCACCT	1827-1832	Wound responsiveness
CoXTH4p	ARE	AAACCA	784-789	Cis-acting regulatory element essential for the anaerobic induction
CoXTH4p	TCCC-motif	TCTCCCT	311-317	Part of a light responsive element
CoXTH4p	WUN-motif	AAATTTCCT	1962-1970	Wound-responsive element
CoXTH4p	GATA-motif	AAGGATAAGG	156-164	Part of a light responsive element
CoXTH4p	ABRE	ACGTG	1771-1775	Cis-acting element involved in the abscisic acid responsiveness

CoXTH4p	Box 4	ATTAAT	435-440	Part of a conserved DNA module involved in light responsiveness
CoXTH4p	Box 4	ATTAAT	707-712	Part of a conserved DNA module involved in light responsiveness
CoXTH4p	AT1-motif	AATTATTTTTTATT	1386-1398	Part of a light responsive module
CoXTH4p	AT1-motif	AATTATTTTTTATT	1849-1861	Part of a light responsive module
CoXTH4p	GT1-motif	GGTTAAT	939-945	Light responsive element
CoXTH4p	STRE	AGGGG	186-190	Osmotic stress response element
CoXTH5p	ABRE	ACGTG	748-752	Cis-acting element involved in the abscisic acid responsiveness
CoXTH5p	chs-CMA2a	TCACTTGA	869-876	Part of a light responsive element
CoXTH5p	ARE	AAACCA	711-716	Cis-acting regulatory element essential for the anaerobic induction
CoXTH5p	MYB	TAACCA	1349-1354	MYB Binding Site
CoXTH5p	MYB	CAACCA	1738-1743	MYB Binding Site
CoXTH5p	as-1	TGACG	1813-1817	Cis-acting element involved in the MeJA responsiveness
CoXTH5p	MYC	CAATTG	432-437	MYC binding site
CoXTH5p	WUN-motif	CCATTTCAA	1862-1870	Stress-responsive element
CoXTH5p	Box 4	ATTAAT	932-937	Part of a conserved DNA module involved in light responsiveness
CoXTH5p	Box 4	ATTAAT	988-993	Part of a conserved DNA module involved in light responsiveness
CoXTH5p	AAGAA-motif	GAAAGAA	1584-1590	Cis-acting element involved in the abscisic acid responsiveness
CoXTH5p	STRE	AGGGG	526-530	Osmotic stress response element
CoXTH5p	STRE	AGGGG	588-592	Osmotic stress response element
CoXTH5p	MYB	CAACTG	131-136	MYB Binding Site
CoXTH5p	MYB	TAACCA	1349-1354	MYB Binding Site
CoXTH5p	CGTCA-motif	CGTCA	119-123	Cis-acting regulatory element involved in the MeJA responsiveness
CoXTH5p	TGACG-motif	TGACG	1813-1817	Cis-acting regulatory element involved in the MeJA responsiveness
CoXTH5p	MBS	CAACTG	131-136	MYB binding site involved in drought-inducibility
CoXTH5p	LTR	CCGAAA	101-106	Cis-acting element involved in low-temperature responsiveness
CoXTH5p	WRE3	CCACCT	350-355	Wound responsiveness

CoXTH5p	WRE3	CCACCT	378-383	Wound responsiveness
CoXTH6p	Box 4	ATTAAT	205-210	Part of a conserved DNA module involved in light responsiveness
CoXTH6p	Box 4	ATTAAT	354-359	Part of a conserved DNA module involved in light responsiveness
CoXTH6p	P-box	CCTTTTG	1078-1084	Gibberellin-responsive element
CoXTH6p	TATC-box	TATCCCA	1090-1096	Cis-acting element involved in gibberellin-responsiveness
CoXTH6p	TATC-box	TATCCCA	1213-1219	Cis-acting element involved in gibberellin-responsiveness
CoXTH6p	TCT-motif	TCTTAC	311-316	Part of a light responsive element
CoXTH6p	MYB	CCGTTG	509-514	MYB binding site
CoXTH6p	WUN-motif	AAATTTCCT	361-369	Wound-responsive element
CoXTH6p	O2-site	GATGATGTGG	1404-1412	Cis-acting regulatory element involved in zein metabolism regulation
CoXTH6p	F-box	CTATTCTCATT	107-116	Gibberellin response element
CoXTH6p	ARE	AAACCA	25-30	Cis-acting regulatory element essential for the anaerobic induction
CoXTH6p	ARE	AAACCA	1500-1505	Cis-acting regulatory element essential for the anaerobic induction
CoXTH6p	ARE	AAACCA	1916-1921	Cis-acting regulatory element essential for the anaerobic induction
CoXTH6p	I-box	AGATAAGG	603-610	Part of a light responsive element
CoXTH6p	GATA-motif	AAGGATAAGG	601-609	Part of a light responsive element
CoXTH6p	STRE	AGGGG	581-585	Osmotic stress response element
CoXTH6p	STRE	AGGGG	608-612	Osmotic stress response element
CoXTH6p	ERE	ATTTTAAA	20-27	Ethylene response element
CoXTH6p	ERE	ATTTTAAA	769-776	Ethylene response element
CoXTH6p	MYB	CAACCA	1123-1128	MYB binding site
CoXTH7p	TC-rich repeats	GTTTTCTTAC	524-532	Cis-acting element involved in defense and stress responsiveness
CoXTH7p	ABRE4	CACGTA	1620-1625	Cis-acting element involved in the abscisic acid responsiveness
CoXTH7p	ABRE4	CACGTA	1673-1678	Cis-acting element involved in the abscisic acid responsiveness
CoXTH7p	MYC	CATTTG	519-524	MYC binding site
CoXTH7p	MYC	CATGTG	1066-1071	MYC binding site

CoXTH7p	MYC	CATGTG	1333-1338	MYC binding site
CoXTH7p	ABRE	ACGTG	678-682	Cis-acting element involved in the abscisic acid responsiveness
CoXTH7p	ABRE	GCAACGTGTC	1224-1232	Cis-acting element involved in the abscisic acid responsiveness
CoXTH7p	ABRE	ACGTG	1227-1231	Cis-acting element involved in the abscisic acid responsiveness
CoXTH7p	ABRE	AACCCGG	1436-1442	Cis-acting element involved in the abscisic acid responsiveness
CoXTH7p	circadian	CAAAGATATC	788-796	Cis-acting regulatory element involved in circadian control
CoXTH7p	MYB	CAACCA	237-242	MYB binding site
CoXTH7p	MBS	CAACTG	902-907	MYB binding site involved in drought-inducibility
CoXTH7p	ERE	ATTTCATA	1825-1832	Ethylene responsive element
CoXTH7p	MYB	CAACTG	902-907	MYB binding site
CoXTH7p	G-box	TAAACGTG	675-682	Cis-acting regulatory element involved in light responsiveness
CoXTH7p	G-box	CACGTC	689-694	Cis-acting regulatory element involved in light responsiveness
CoXTH7p	MYB	CCGTTG	1160-1165	MYB binding site
CoXTH7p	ARE	AAACCA	257-262	Cis-acting regulatory element essential for the anaerobic induction
CoXTH7p	ACE	CTAACGTATT	1618-1626	Cis-acting element involved in light responsiveness
CoXTH7p	WUN-motif	AAATTTCCT	1904-1912	Wound-responsive element
CoXTH7p	ATCT-motif	AATCTAATCC	1080-1088	Part of a conserved DNA module involved in light responsiveness
CoXTH7p	Box 4	ATTAAT	17-22	Part of a conserved DNA module involved in light responsiveness
CoXTH7p	Box 4	ATTAAT	647-652	Part of a conserved DNA module involved in light responsiveness
CoXTH7p	Box 4	ATTAAT	763-768	Part of a conserved DNA module involved in light responsiveness
CoXTH7p	Box 4	ATTAAT	801-806	Part of a conserved DNA module involved in light responsiveness
CoXTH8p	GT1-motif	GGTTAAT	851-857	Light responsive element
CoXTH8p	G-Box	CACGTT	166-171	Cis-acting regulatory element involved in light responsiveness
CoXTH8p	CCGTCC-box	CCGTCC	1503-1508	Cis-acting regulatory element related to fruit development
CoXTH8p	ARE	AAACCA	284-289	Cis-acting regulatory element essential for the anaerobic induction
CoXTH8p	TCA-element	CCATCTTTTT	1613-1621	Cis-acting element involved in salicylic acid responsiveness

CoXTH8p	MYC	CAATTG	470-475	MYC binding site
CoXTH8p	MYC	CATTTG	562-567	MYC binding site
CoXTH8p	MYC	CATTTG	663-668	MYC binding site
CoXTH8p	MYC	CATTTG	1328-1333	MYC binding site
CoXTH8p	LAMP-element	CTTTATCA	955-962	Part of a light responsive element
CoXTH8p	MYB	CAACCA	1649-1654	MYB binding site
CoXTH8p	Box 4	ATTAAT	18-23	Part of a conserved DNA module involved in light responsiveness
CoXTH8p	Box 4	ATTAAT	67-72	Part of a conserved DNA module involved in light responsiveness
CoXTH8p	Box 4	ATTAAT	326-331	Part of a conserved DNA module involved in light responsiveness
CoXTH8p	AAGAA-motif	GAAAGAA	963-969	Cis-acting element involved in the abscisic acid responsiveness
CoXTH8p	AAGAA-motif	GAAAGAA	1541-1547	Cis-acting element involved in the abscisic acid responsiveness
CoXTH8p	TCT-motif	TCTTAC	1738-1743	Part of a light responsive element
CoXTH8p	ATCT-motif	AATCTAATCC	289-297	Part of a conserved DNA module involved in light responsiveness
CoXTH8p	ERE	ATTTTAAA	175-182	Ethylene response element
CoXTH8p	as-1	TGACG	1016-1020	Cis-acting element involved in the MeJA responsiveness
CoXTH8p	TGACG-motif	TGACG	1016-1020	Cis-acting regulatory element involved in the MeJA responsiveness
CoXTH8p	WRE3	CCACCT	686-691	Wound responsiveness
CoXTH8p	STRE	AGGGG	317-321	Osmotic stress response element
CoXTH9p	MYB	TAACCA	1167-1172	MYB binding site
CoXTH9p	MYB	TAACTG	660-665	MYB binding site
CoXTH9p	MYB	TAACTG	1224-1229	MYB binding site
CoXTH9p	TCT-motif	TCTTAC	202-207	Part of a light responsive element
CoXTH9p	LTR	CCGAAA	451-456	Cis-acting element involved in low-temperature responsiveness
CoXTH9p	ERE	ATTTCATA	23-30	Ethylene response element
CoXTH9p	ERE	ATTTCATA	109-116	Ethylene response element
CoXTH9p	MYC	TCTCTTA	655-661	MYC binding site

CoXTH9p	MYC	TCTCTTA	1462-1468	MYC binding site
CoXTH9p	MRE	AACCTAA	706-712	MYB binding site involved in light responsiveness
CoXTH9p	CAT-box	GCCACT	324-329	Cis-acting regulatory element related to meristem expression
CoXTH9p	CAT-box	GCCACT	405-410	Cis-acting regulatory element related to meristem expression
CoXTH9p	MYB	TAACCA	1167-1172	MYB binding site
CoXTH9p	P-box	CCTTTTG	1112-1118	Gibberellin-responsive element
CoXTH9p	AAGAA-motif	GGTAAAGAAA	549-557	Cis-acting element involved in the abscisic acid responsiveness
CoXTH9p	MYC	CATGTG	1616-1621	MYC binding site
CoXTH9p	GT1-motif	GGTTAA	858-863	Light responsive element
CoXTH9p	GT1-motif	GGTTAA	1945-1950	Light responsive element
CoXTH10p	ACE	CTAACGTATT	740-748	Cis-acting element involved in light responsiveness
CoXTH10p	CGTCA-motif	CGTCA	1833-1837	Cis-acting regulatory element involved in the MeJA responsiveness
CoXTH10p	WRE3	CCACCT	1852-1857	Wound responsiveness
CoXTH10p	WRE3	CCACCT	1946-1951	Wound responsiveness
CoXTH10p	W box	TTGACC	1979-1984	Stress-responsive element
CoXTH10p	AAGAA-motif	GGTAAAGAAA	948-956	Cis-acting element involved in the abscisic acid responsiveness
CoXTH10p	ABRE4	CACGTA	269-274	Cis-acting element involved in the abscisic acid responsiveness
CoXTH10p	ABRE4	CACGTA	742-747	Cis-acting element involved in the abscisic acid responsiveness
CoXTH10p	MRE	AACCTAA	822-828	MYB binding site involved in light responsiveness
CoXTH10p	TGACG-motif	TGACG	1146-1150	Cis-acting regulatory element involved in the MeJA responsiveness
CoXTH10p	Box 4	ATTAAT	726-731	Part of a conserved DNA module involved in light responsiveness
CoXTH10p	Box 4	ATTAAT	730-735	Part of a conserved DNA module involved in light responsiveness
CoXTH10p	Box 4	ATTAAT	864-869	Part of a conserved DNA module involved in light responsiveness
CoXTH10p	MBS	CAACTG	1123-1128	MYB binding site involved in drought-inducibility
CoXTH10p	MYB	CAACTG	1123-1128	MYB binding site
CoXTH10p	MYB	CAACCA	1849-1854	MYB binding site

CoXTH10p	ARE	AAACCA	1490-1495	Cis-acting regulatory element essential for the anaerobic induction
CoXTH10p	ARE	AAACCA	1918-1923	Cis-acting regulatory element essential for the anaerobic induction
CoXTH10p	ATC-motif	AGTAATCT	511-518	Part of a conserved DNA module involved in light responsiveness
CoXTH10p	ATC-motif	TGCTATCCA	678-686	Part of a conserved DNA module involved in light responsiveness
CoXTH10p	chs-CMA1a	TTACTTAA	286-293	Part of a light responsive element
CoXTH10p	as-1	TGACG	1146-1150	Cis-acting element involved in the MeJA responsiveness
CoXTH11p	ARE	AAACCA	540-545	Cis-acting regulatory element essential for the anaerobic induction
CoXTH11p	ARE	AAACCA	545-550	Cis-acting regulatory element essential for the anaerobic induction
CoXTH11p	ARE	AAACCA	762-767	Cis-acting regulatory element essential for the anaerobic induction
CoXTH11p	ARE	AAACCA	908-913	Cis-acting regulatory element essential for the anaerobic induction
CoXTH11p	ARE	AAACCA	1727-1732	Cis-acting regulatory element essential for the anaerobic induction
CoXTH11p	I-box	AAGATAAGGCT	987-996	Part of a light responsive element
CoXTH11p	MYB	CAACCA	941-946	MYB binding site
CoXTH11p	STRE	AGGGG	854-858	Osmotic stress response element
CoXTH11p	STRE	AGGGG	1238-1242	Osmotic stress response element
CoXTH11p	MYC	CATGTG	630-635	MYC binding site
CoXTH11p	WUN-motif	CAATTACAT	14-22	Stress-responsive element
CoXTH11p	WUN-motif	AAATTTCTT	699-707	Stress-responsive element
CoXTH11p	ERE	ATTTTAAA	460-467	Ethylene response element
CoXTH12p	LAMP-element	CTTTATCA	431-438	Part of a light responsive element
CoXTH12p	MRE	AACCTAA	181-187	MYB binding site involved in light responsiveness
CoXTH12p	STRE	AGGGG	86-90	Osmotic stress response element
CoXTH12p	MYB	TAACCA	809-814	MYB binding site
CoXTH12p	MYC	CATGTG	1625-1630	MYC binding site
CoXTH12p	GATA-motif	AAGGATAAGG	97-105	Part of a light responsive element
CoXTH12p	MYC	TCTCTTA	775-781	MYC binding site

CoXTH12p	ABRE	ACGTG	452-456	Cis-acting element involved in the abscisic acid responsiveness
CoXTH12p	MYB	CAACAG	279-284	MYB binding site
CoXTH12p	MYB	TAACCA	809-814	MYB binding site
CoXTH12p	AAGAA-motif	GGTAAAGAAA	366-374	Cis-acting element involved in the abscisic acid responsiveness
CoXTH12p	AAGAA-motif	GAAAGAA	368-374	Cis-acting element involved in the abscisic acid responsiveness
CoXTH12p	ARE	AAACCA	696-701	Cis-acting regulatory element essential for the anaerobic induction
CoXTH12p	ARE	AAACCA	1355-1360	Cis-acting regulatory element essential for the anaerobic induction
CoXTH12p	ARE	AAACCA	1850-1855	Cis-acting regulatory element essential for the anaerobic induction
CoXTH12p	ARE	AAACCA	1952-1957	Cis-acting regulatory element essential for the anaerobic induction
CoXTH12p	Box 4	ATTAAT	349-354	Part of a conserved DNA module involved in light responsiveness
CoXTH12p	O2-site	GATGA(C/T)(A/G)TG(A/G)	1213-1220	Cis-acting regulatory element involved in zein metabolism regulation
CoXTH12p	chs-CMA1a	TTACTTAA	1681-1688	Part of a light responsive element
CoXTH12p	I-box	AAGATAAGGCT	657-666	Part of a light responsive element
CoXTH12p	TCT-motif	TCTTAC	613-618	Part of a light responsive element
CoXTH12p	TCT-motif	TCTTAC	777-782	Part of a light responsive element
CoXTH12p	MYB	CAACAG	279-284	MYB binding site
CoXTH12p	chs-CMA2a	TCACTTGA	67-74	Part of a light responsive element
CoXTH13p	TCCC-motif	TCTCCCT	1442-1448	Part of a light responsive element
CoXTH13p	TCCC-motif	TCTCCCT	1452-1458	Part of a light responsive element
CoXTH13p	TCCC-motif	TCTCCCT	1488-1494	Part of a light responsive element
CoXTH13p	TC-rich repeats	GTTTTCTTAC	1577-1585	Cis-acting element involved in defense and stress responsiveness
CoXTH13p	ARE	AAACCA	1661-1666	Cis-acting regulatory element essential for the anaerobic induction
CoXTH13p	ARE	AAACCA	1919-1924	Cis-acting regulatory element essential for the anaerobic induction
CoXTH13p	ERE	ATTTTAAA	1014-1021	Ethylene response element
CoXTH13p	CAT-box	GCCACT	2-7	Cis-acting regulatory element related to meristem expression
CoXTH13p	MYB	TAACTG	1572-1577	MYB binding site

CoXTH13p	WRE3	CCACCT	43-48	Wound responsiveness
CoXTH13p	G-box	TAAACGTG	1121-1128	Cis-acting regulatory element involved in light responsiveness
CoXTH13p	3-AF1 binding site	TAAGAGAGGAA	1953-1962	Light responsive element
CoXTH13p	MRE	AACCTAA	1679-1685	MYB binding site involved in light responsiveness
CoXTH13p	MYC	CATTTG	330-335	MYC binding site
CoXTH13p	MYC	CATTTG	1348-1353	MYC binding site
CoXTH13p	MYC	CATTTG	1886-1891	MYC binding site
CoXTH13p	G-Box	CACGTT	473-478	Cis-acting regulatory element involved in light responsiveness
CoXTH13p	ABRE	ACGTG	1124-1128	Cis-acting element involved in the abscisic acid responsiveness
CoXTH13p	GATA-motif	AAGATAAGATT	505-514	Part of a light responsive element
CoXTH13p	DRE core	GCCGAC	92-97	Dehydration response element TC-rich repeats
CoXTH13p	circadian	CAAAGATATC	503-511	Cis-acting regulatory element involved in circadian control
CoXTH13p	WUN-motif	CCATTTCAA	1939-1947	Stress-responsive element
CoXTH13p	GT1-motif	GGTTAA	599-604	Light responsive element
CoXTH13p	GT1-motif	GGTTAA	1818-1823	Light responsive element
CoXTH13p	LTR	CCGAAA	1675-1680	Cis-acting element involved in low-temperature responsiveness
CoXTH13p	TCT-motif	TCTTAC	1581-1586	Part of a light responsive element
CoXTH14p	MYB	TAACCA	160-165	MYB binding site involved in drought-inducibility
CoXTH14p	G-box	TACGTG	718-723	Cis-acting regulatory element involved in light responsiveness
CoXTH14p	MYC	CAATTG	25-30	MYC binding site
CoXTH14p	MYC	CAATTG	447-452	MYC binding site
CoXTH14p	MYC	CAATTG	497-502	MYC binding site
CoXTH14p	MYC	CATGTG	586-591	MYC binding site
CoXTH14p	MYB	CCGTTG	539-544	MYB binding site involved in drought-inducibility
CoXTH14p	MYB	CCGTTG	1404-1409	MYB binding site involved in drought-inducibility
CoXTH14p	STRE	AGGGG	636-640	Osmotic stress response element

CoXTH14p	STRE	AGGGG	1349-1353	Osmotic stress response element
CoXTH14p	Box 4	ATTAAT	197-202	Part of a conserved DNA module involved in light responsiveness
CoXTH14p	Box 4	ATTAAT	215-220	Part of a conserved DNA module involved in light responsiveness
CoXTH14p	Box 4	ATTAAT	268-273	Part of a conserved DNA module involved in light responsiveness
CoXTH14p	Box 4	ATTAAT	389-394	Part of a conserved DNA module involved in light responsiveness
CoXTH14p	TCT-motif	TCTTAC	994-999	Part of a light responsive element
CoXTH14p	GT1-motif	GTGTGTGAA	707-715	Light responsive element
CoXTH14p	GT1-motif	GGTTAA	1104-1109	Light responsive element
CoXTH14p	MYB	TAACTG	1024-1029	MYB binding site involved in drought-inducibility
CoXTH14p	ABRE3a	TACGTG	718-723	Cis-acting element involved in the abscisic acid responsiveness
CoXTH14p	GC-motif	CCCCCG	1778-1783	Enhancer-like element involved in anoxic specific inducibility
CoXTH14p	W box	TTGACC	39-44	Stress-responsive element
CoXTH14p	CGTCA-motif	CGTCA	81-85	Cis-acting regulatory element involved in the MeJA responsiveness
CoXTH14p	MYB	TAACCA	160-165	MYB binding site involved in drought-inducibility
CoXTH14p	MYB	CAACCA	1611-1616	MYB binding site involved in drought-inducibility
CoXTH14p	MYB	CAACCA	1677-1682	MYB binding site involved in drought-inducibility
CoXTH14p	MYB	CAACCA	1983-1988	MYB binding site involved in drought-inducibility
CoXTH14p	ABRE	ACGTG	719-723	Cis-acting element involved in the abscisic acid responsiveness
CoXTH14p	ARE	AAACCA	1316-1321	Cis-acting regulatory element essential for the anaerobic induction
CoXTH14p	ARE	AAACCA	1583-1588	Cis-acting regulatory element essential for the anaerobic induction
CoXTH14p	GATA-motif	AAGATAAGATT	169-178	Part of a light responsive element
CoXTH14p	WRE3	CCACCT	1469-1474	Wound responsiveness
CoXTH14p	ERE	ATTTTAAA	134-141	Ethylene response element
CoXTH14p	ERE	ATTTTAAA	219-226	Ethylene response element
CoXTH14p	TCCC-motif	TCTCCCT	1864-1870	Part of a light responsive element
CoXTH14p	TCCC-motif	TCTCCCT	1874-1880	Part of a light responsive element

CoXTH15p	W box	TTGACC	1070-1075	Stress-responsive element
CoXTH15p	STRE	AGGGG	65-69	Osmotic stress response element
CoXTH15p	STRE	AGGGG	211-215	Osmotic stress response element
CoXTH15p	STRE	AGGGG	239-243	Osmotic stress response element
CoXTH15p	STRE	AGGGG	267-271	Osmotic stress response element
CoXTH15p	LTR	CCGAAA	35-40	Cis-acting element involved in low-temperature responsiveness
CoXTH15p	CGTCA-motif	CGTCA	1023-1027	Cis-acting regulatory element involved in the MeJA responsiveness
CoXTH15p	CAT-box	GCCACT	1253-1258	Cis-acting regulatory element related to meristem expression
CoXTH15p	DRE core	GCCGAC	617-622	Dehydration response element TC-rich repeats
CoXTH15p	DRE core	GCCGAC	656-661	Dehydration response element TC-rich repeats
CoXTH15p	DRE core	GCCGAC	708-713	Dehydration response element TC-rich repeats
CoXTH15p	chs-CMA1a	TTACTTAA	1577-1584	Part of a light responsive element
CoXTH15p	GCN4_motif	TGAGTCA	730-736	Cis-regulatory element involved in endosperm expression
CoXTH15p	MYB	TAACCA	1950-1955	MYB binding site involved in drought-inducibility
CoXTH15p	Box 4	ATTAAT	982-987	Part of a conserved DNA module involved in light responsiveness
CoXTH15p	Box 4	ATTAAT	986-991	Part of a conserved DNA module involved in light responsiveness
CoXTH15p	GT1-motif	GGTTAA	1041-1046	Light responsive element
CoXTH15p	MYB	TAACCA	1950-1955	MYB binding site involved in drought-inducibility
CoXTH15p	MYC	CATTTG	1052-1057	MYC binding site
CoXTH15p	MYC	CATTTG	1390-1395	MYC binding site
CoXTH15p	MYC	TCTCTTA	1617-1623	MYC binding site
CoXTH15p	MBSI	TTTTTACGGTTA	1034-1044	MYB binding site involved in flavonoid biosynthetic genes regulation
CoXTH15p	TGACG-motif	TGACG	490-494	Cis-acting regulatory element involved in the MeJA responsiveness
CoXTH15p	TGACG-motif	TGACG	642-646	Cis-acting regulatory element involved in the MeJA responsiveness
CoXTH15p	TGACG-motif	TGACG	1037-1041	Cis-acting regulatory element involved in the MeJA responsiveness
CoXTH15p	as-1	TGACG	490-494	Cis-acting element involved in the MeJA responsiveness

CoXTH15p	as-1	TGACG	642-646	Cis-acting element involved in the MeJA responsiveness
CoXTH15p	as-1	TGACG	1037-1041	Cis-acting element involved in the MeJA responsiveness
CoXTH15p	AAGAA-motif	GAAAGAA	23-29	Cis-acting element involved in the abscisic acid responsiveness
CoXTH15p	AAGAA-motif	GAAAGAA	71-77	Cis-acting element involved in the abscisic acid responsiveness
CoXTH16p	WUN-motif	AAATTACT	1529-1536	Stress-responsive element
CoXTH16p	MRE	AACCTAA	998-1004	MYB binding site involved in light responsiveness
CoXTH16p	chs-CMA1a	TTACTTAA	1607-1614	Part of a light responsive element
CoXTH16p	STRE	AGGGG	1281-1285	Osmotic stress response element
CoXTH16p	TCT-motif	TCTTAC	1754-1759	Part of a light responsive element
CoXTH16p	MYB	CAACTG	1771-1776	MYB binding site
CoXTH16p	WRE3	CCACCT	1179-1184	Wound responsiveness
CoXTH16p	MYC	CATGTG	924-929	MYC binding site
CoXTH16p	Box 4	ATTAAT	126-131	Part of a conserved DNA module involved in light responsiveness
CoXTH16p	GT1-motif	GGTTAA	756-761	Light responsive element
CoXTH16p	GT1-motif	GGTTAA	1039-1044	Light responsive element
CoXTH16p	MYB	TAACCA	561-566	MYB binding site
CoXTH16p	MYB	TAACCA	628-633	MYB binding site
CoXTH16p	GATA-motif	AAGGATAAGG	1037-1045	Part of a light responsive element
CoXTH16p	MBS	CAACTG	1771-1776	MYB binding site involved in drought-inducibility
CoXTH16p	MYB	TAACCA	561-566	MYB binding site
CoXTH16p	MYB	TAACCA	628-633	MYB binding site
CoXTH16p	ATC-motif	AGTAATCT	1909-1916	Part of a conserved DNA module involved in light responsiveness
CoXTH16p	ERE	ATTTTAAA	854-861	Ethylene response element
CoXTH16p	ERE	ATTTTAAA	1538-1545	Ethylene response element
CoXTH16p	ARE	AAACCA	533-538	Cis-acting regulatory element essential for the anaerobic induction
CoXTH16p	ARE	AAACCA	538-543	Cis-acting regulatory element essential for the anaerobic induction

CoXTH16p	ARE	AAACCA	543-548	Cis-acting regulatory element essential for the anaerobic induction
CoXTH16p	ARE	AAACCA	1176-1181	Cis-acting regulatory element essential for the anaerobic induction
CoXTH17p	MYB	CAACAG	214-219	MYB binding site
CoXTH17p	MYB	TAACCA	559-564	MYB binding site
CoXTH17p	MYB	TAACCA	625-630	MYB binding site
CoXTH17p	Box 4	ATTAAT	128-133	Part of a conserved DNA module involved in light responsiveness
CoXTH17p	MRE	AACCTAA	984-990	MYB binding site involved in light responsiveness
CoXTH17p	MYB	TAACTG	1955-1960	MYB binding site
CoXTH17p	GCN4_motif	TGAGTCA	901-907	Cis-regulatory element involved in endosperm expression
CoXTH17p	ERE	ATTTTAAA	49-56	Ethylene response element
CoXTH17p	ERE	ATTTTAAA	837-844	Ethylene response element
CoXTH17p	ARE	AAACCA	531-536	Cis-acting regulatory element essential for the anaerobic induction
CoXTH17p	ARE	AAACCA	541-546	Cis-acting regulatory element essential for the anaerobic induction
CoXTH17p	as-1	TGACG	648-652	Cis-acting element involved in the MeJA responsiveness
CoXTH17p	TGACG-motif	TGACG	648-652	Cis-acting regulatory element involved in the MeJA responsiveness
CoXTH17p	MYB	TAACCA	559-564	MYB binding site
CoXTH17p	MYB	TAACCA	625-630	MYB binding site
CoXTH17p	TCT-motif	TCTTAC	1938-1943	Part of a light responsive element
CoXTH17p	MYB	CAACAG	214-219	MYB binding site
CoXTH17p	STRE	AGGGG	1023-1027	Osmotic stress response element
CoXTH17p	STRE	AGGGG	1313-1317	Osmotic stress response element
CoXTH17p	MYC	CATGTG	159-164	MYC binding site
CoXTH17p	MYC	CAATTG	744-749	MYC binding site
CoXTH17p	MYC	CATGTG	897-902	MYC binding site
CoXTH17p	GATA-motif	AAGATAAGATT	74-83	Part of a light responsive element
CoXTH17p	GATA-motif	AAGATAAGATT	442-451	Part of a light responsive element

CoXTH18p	ERE	ATTTTAAA	679-686	Ethylene response element
CoXTH18p	ERE	ATTTCATA	1290-1297	Ethylene response element
CoXTH18p	STRE	AGGGG	761-765	Osmotic stress response element
CoXTH18p	STRE	AGGGG	936-940	Osmotic stress response element
CoXTH18p	STRE	AGGGG	1488-1492	Osmotic stress response element
CoXTH18p	ABRE3a	TACGTG	1859-1864	Cis-acting element involved in the abscisic acid responsiveness
CoXTH18p	AAGAA-motif	GGTAAAGAAA	667-675	Cis-acting element involved in the abscisic acid responsiveness
CoXTH18p	MBS	CAACTG	1639-1644	MYB binding site involved in drought-inducibility
CoXTH18p	as-1	TGACG	1870-1874	Cis-acting element involved in the MeJA responsiveness
CoXTH18p	ABRE	ACGTG	1674-1678	Cis-acting element involved in the abscisic acid responsiveness
CoXTH18p	ABRE	ACGTG	1860-1864	Cis-acting element involved in the abscisic acid responsiveness
CoXTH18p	ABRE	ACGTG	1872-1876	Cis-acting element involved in the abscisic acid responsiveness
CoXTH18p	G-box	TACGTG	1859-1864	Cis-acting regulatory element involved in light responsiveness
CoXTH18p	TGACG-motif	TGACG	1870-1874	Cis-acting regulatory element involved in the MeJA responsiveness
CoXTH18p	MYB	TAACCA	366-371	MYB binding site involved in drought-inducibility
CoXTH18p	MYB	CAACCA	809-814	MYB binding site involved in drought-inducibility
CoXTH18p	MYC	CAATTG	99-104	MYC binding site
CoXTH18p	MYB	TAACTG	1445-1450	MYB binding site involved in drought-inducibility
CoXTH18p	MYB	CAACTG	1639-1644	MYB binding site involved in drought-inducibility
CoXTH18p	O2-site	GATGATGTGG	440-448	Cis-acting regulatory element involved in zein metabolism regulation
CoXTH18p	O2-site	GATGATGTGG	443-451	Cis-acting regulatory element involved in zein metabolism regulation
CoXTH18p	TC-rich repeats	ATTCTCTAAC	331-339	Cis-acting element involved in defense and stress responsiveness
CoXTH18p	Box II	CCACGTGGC	1672-1680	Part of a light responsive element
CoXTH18p	MYB	TAACCA	366-371	MYB binding site involved in drought-inducibility
CoXTH18p	CCAAT-box	CAACGG	1809-1814	MYBHv1 binding site
CoXTH19p	Box 4	ATTAAT	867-872	Part of a conserved DNA module involved in light responsiveness

CoXTH19p	Box 4	ATTAAT	941-946	Part of a conserved DNA module involved in light responsiveness
CoXTH19p	MYB	TAACCA	815-820	MYB binding site involved in drought-inducibility
CoXTH19p	MYC	CAATTG	613-618	MYC binding site
CoXTH19p	MYC	CATTTG	1729-1734	MYC binding site
CoXTH19p	WUN-motif	AAATTTCTT	135-143	Wound-responsive element
CoXTH19p	WUN-motif	AAATTTCCT	441-449	Wound-responsive element
CoXTH19p	ERE	ATTTTAAA	684-691	Ethylene response element
CoXTH19p	ERE	ATTTTAAA	1101-1108	Ethylene response element
CoXTH19p	ERE	ATTTTAAA	1370-1377	Ethylene response element
CoXTH19p	ATCT-motif	AATCTAATCC	654-662	Part of a conserved DNA module involved in light responsiveness
CoXTH19p	MYB	TAACCA	815-820	MYB binding site involved in drought-inducibility
CoXTH19p	LTR	CCGAAA	1503-1508	Cis-acting element involved in low-temperature responsiveness
CoXTH19p	GT1-motif	GGTTAA	717-722	Light responsive element
CoXTH19p	AuxRR-core	GGTCCAT	479-485	Cis-acting regulatory element involved in auxin responsiveness
CoXTH20p	TC-rich repeats	GTTTTCTTAC	1172-1180	Cis-acting element involved in defense and stress responsiveness
CoXTH20p	TC-rich repeats	ATTCTCTAAC	1814-1822	Cis-acting element involved in defense and stress responsiveness
CoXTH20p	G-Box	CACGTT	1127-1132	Cis-acting regulatory element involved in light responsiveness
CoXTH20p	GARE-motif	TCTGTTG	1288-1294	Gibberellin-responsive element
CoXTH20p	MYC	CATTTG	1006-1011	MYC binding site
CoXTH20p	MYC	CATTTG	1784-1789	MYC binding site
CoXTH20p	MYB	CAACCA	516-521	MYB binding site involved in drought-inducibility
CoXTH20p	MYB	TAACCA	1498-1503	MYB binding site involved in drought-inducibility
CoXTH20p	STRE	AGGGG	1201-1205	Osmotic stress response element
CoXTH20p	MYB	TAACCA	1498-1503	MYB binding site involved in drought-inducibility
CoXTH20p	ERE	ATTTTAAA	120-127	Ethylene response element
CoXTH20p	ERE	ATTTTAAA	373-380	Ethylene response element

CoXTH20p	ERE	ATTTTAAA	1388-1395	Ethylene response element
CoXTH20p	AAAC-motif	CAATCAAAACCT	1114-1124	Light responsive element
CoXTH21p	CCGTCC-box	CCGTCC	537-542	Cis-acting regulatory element related to fruit development
CoXTH21p	MYB	TAACCA	866-871	MYB binding site involved in drought-inducibility
CoXTH21p	MYB	TAACCA	1366-1371	MYB binding site involved in drought-inducibility
CoXTH21p	MYC	CATTTG	350-355	MYC binding site
CoXTH21p	ATCT-motif	AATCTAATCC	421-429	Part of a conserved DNA module involved in light responsiveness
CoXTH21p	WUN-motif	TAATTACTC	705-713	Stress-responsive element
CoXTH21p	Box 4	ATTAAT	700-705	Part of a conserved DNA module involved in light responsiveness
CoXTH21p	ARE	AAACCA	102-107	Cis-acting regulatory element essential for the anaerobic induction
CoXTH21p	MYB	TAACCA	866-871	MYB binding site involved in drought-inducibility
CoXTH21p	MYB	TAACCA	1366-1371	MYB binding site involved in drought-inducibility
CoXTH21p	STRE	AGGGG	947-951	Osmotic stress response element
CoXTH21p	TATC-box	TATCCCA	377-383	Cis-acting element involved in gibberellin-responsiveness
CoXTH21p	TATC-box	TATCCCA	489-495	Cis-acting element involved in gibberellin-responsiveness
CoXTH22p	WUN-motif	TTATTACAT	817-825	Stress-responsive element
CoXTH22p	TCA-element	CCATCTTTTT	1321-1329	Cis-acting element involved in salicylic acid responsiveness
CoXTH22p	Box 4	ATTAAT	605-610	Part of a conserved DNA module involved in light responsiveness
CoXTH22p	Box 4	ATTAAT	673-678	Part of a conserved DNA module involved in light responsiveness
CoXTH22p	Box 4	ATTAAT	748-753	Part of a conserved DNA module involved in light responsiveness
CoXTH22p	ARE	AAACCA	1732-1737	Cis-acting regulatory element essential for the anaerobic induction
CoXTH22p	MYB	CAACTG	417-422	MYB binding site involved in drought-inducibility
CoXTH22p	MBS	CAACTG	417-422	MYB binding site involved in drought-inducibility
CoXTH22p	MYC	CATGTG	989-994	MYC binding site
CoXTH22p	MYC	CATGTG	1205-1210	MYC binding site
CoXTH22p	MYC	CATTTG	1338-1343	MYC binding site

CoXTH22p	G-Box	CACGTT	184-189	Cis-acting regulatory element involved in light responsiveness
CoXTH22p	G-Box	CACGTT	1068-1073	Cis-acting regulatory element involved in light responsiveness
CoXTH22p	ERE	ATTTTAAA	245-252	Ethylene response element
CoXTH22p	ERE	ATTTCATA	692-699	Ethylene response element
CoXTH22p	ERE	ATTTTAAA	713-720	Ethylene response element
CoXTH22p	ERE	ATTTTAAA	1538-1545	Ethylene response element
CoXTH22p	WRE3	CCACCT	1946-1951	Wound responsiveness
CoXTH22p	STRE	AGGGG	27-31	Osmotic stress response element
CoXTH22p	AAGAA-motif	GAAAGAA	1408-1414	Cis-acting element involved in the abscisic acid responsiveness
CoXTH22p	CCAAT-box	CAACGG	231-236	MYBHv1 binding site
CoXTH22p	CAT-box	GCCACT	205-210	Cis-acting regulatory element related to meristem expression
CoXTH23p	G-box	GCCACGTGGA	352-360	Cis-acting regulatory element involved in light responsiveness
CoXTH23p	G-box	ACACGTG(G/t)CACC	353-362.5	Cis-acting regulatory element involved in light responsiveness
CoXTH23p	G-box	CACGTG	354-359	Cis-acting regulatory element involved in light responsiveness
CoXTH23p	AE-box	AGAAACAA	1940-1947	Part of a module for light response
CoXTH23p	MYC	CATGTG	393-398	MYC binding site
CoXTH23p	MYC	CATGTG	758-763	MYC binding site
CoXTH23p	MYC	CATTTG	1149-1154	MYC binding site
CoXTH23p	MYC	CATTTG	1410-1415	MYC binding site
CoXTH23p	G-Box	CACGTG	354-359	Cis-acting regulatory element involved in light responsiveness
CoXTH23p	as-1	TGACG	624-628	Cis-acting element involved in the MeJA responsiveness
CoXTH23p	I-box	TAGATAACC	307-315	Part of a light responsive element
CoXTH23p	ABRE	GCCGCGTGGC	352-360	Cis-acting element involved in the abscisic acid responsiveness
CoXTH23p	ABRE	CACGTG	354-359	Cis-acting element involved in the abscisic acid responsiveness
CoXTH23p	ABRE	ACGTG	355-359	Cis-acting element involved in the abscisic acid responsiveness
CoXTH23p	GATA-motif	GATAGGG	201-207	Part of a light responsive element

CoXTH23p	O2-site	GTTGACGTGA	1804-1812	Cis-acting regulatory element involved in zein metabolism regulation
CoXTH23p	Box II	CCACGTGGC	353-361	Part of a light responsive element
CoXTH23p	ERE	ATTTTAAA	909-916	Ethylene response element
CoXTH23p	TGACG-motif	TGACG	624-628	Cis-acting regulatory element involved in the MeJA responsiveness
CoXTH23p	TCA-element	CCATCTTTTT	1924-1932	Cis-acting element involved in salicylic acid responsiveness
CoXTH23p	MYB	TAACCA	322-327	MYB binding site involved in drought-inducibility
CoXTH23p	CGTCA-motif	CGTCA	340-344	Cis-acting regulatory element involved in the MeJA responsiveness
CoXTH23p	CGTCA-motif	CGTCA	381-385	Cis-acting regulatory element involved in the MeJA responsiveness
CoXTH23p	WUN-motif	AAATTACTA	1113-1121	Stress-responsive element
CoXTH23p	ABRE2	CCACGTGG	353-360	Cis-acting element involved in the abscisic acid responsiveness
CoXTH23p	chs-CMA2a	TCACTTGA	662-669	Part of a light responsive element
CoXTH23p	MYB	TAACCA	322-327	MYB binding site involved in drought-inducibility
CoXTH23p	STRE	AGGGG	802-806	Osmotic stress response element
CoXTH23p	STRE	AGGGG	816-820	Osmotic stress response element
CoXTH23p	STRE	AGGGG	828-832	Osmotic stress response element
CoXTH23p	TCT-motif	TCTTAC	1546-1551	Part of a light responsive element
CoXTH23p	Box 4	ATTAAT	523-528	Part of a conserved DNA module involved in light responsiveness
CoXTH23p	GA-motif	ATAGATAA	1485-1492	Part of a light responsive element
CoXTH24p	GARE-motif	TCTGTTG	1110-1116	Gibberellin-responsive element
CoXTH24p	GATA-motif	GATAGGA	1225-1231	Part of a light responsive element
CoXTH24p	F-box	CTATTCTCATT	1522-1531	Gibberellin response element
CoXTH24p	STRE	AGGGG	1730-1734	Osmotic stress response element
CoXTH24p	circadian	CAAAGATATC	824-832	Cis-acting regulatory element involved in circadian control
CoXTH24p	MYB	TAACCA	841-846	MYB binding site involved in drought-inducibility
CoXTH24p	CGTCA-motif	CGTCA	295-299	Cis-acting regulatory element involved in the MeJA responsiveness
CoXTH24p	CGTCA-motif	CGTCA	413-417	Cis-acting regulatory element involved in the MeJA responsiveness

CoXTH24p	TATC-box	TATCCCA	912-918	Cis-acting element involved in gibberellin-responsiveness
CoXTH24p	MRE	AACCTAA	340-346	MYB binding site involved in light responsiveness
CoXTH24p	MYB	TAACCA	841-846	MYB binding site involved in drought-inducibility
CoXTH24p	WUN-motif	CAATTACAT	657-665	Stress-responsive element
CoXTH24p	GT1-motif	GGTTAA	451-456	Light responsive element
CoXTH24p	MYB	CAACTG	712-717	MYB binding site involved in drought-inducibility
CoXTH24p	MYB	CAACTG	1807-1812	MYB binding site involved in drought-inducibility
CoXTH24p	as-1	TGACG	410-414	Cis-acting element involved in the MeJA responsiveness
CoXTH24p	MYC	CATTTG	183-188	MYC binding site
CoXTH24p	MYC	CATTTG	219-224	MYC binding site
CoXTH24p	MYC	CATGTG	947-952	MYC binding site
CoXTH24p	MYC	CATTTG	1741-1746	MYC binding site
CoXTH24p	MYC	TCTCTTA	907-913	MYC binding site
CoXTH24p	MYC	TCTCTTA	1652-1658	MYC binding site
CoXTH24p	TGACG-motif	TGACG	410-414	Cis-acting regulatory element involved in the MeJA responsiveness
CoXTH24p	O2-site	GATGATGTGG	1941-1949	Cis-acting regulatory element involved in zein metabolism regulation
CoXTH24p	MBS	CAACTG	712-717	MYB binding site involved in drought-inducibility
CoXTH24p	MBS	CAACTG	1807-1812	MYB binding site involved in drought-inducibility
CoXTH24p	ARE	AAACCA	1325-1330	Cis-acting regulatory element essential for the anaerobic induction
CoXTH25p	WUN-motif	TTATTACAT	1124-1132	Stress-responsive element
CoXTH25p	STRE	AGGGG	636-640	Osmotic stress response element
CoXTH25p	Box 4	ATTAAT	240-245	Part of a conserved DNA module involved in light responsiveness
CoXTH25p	as-1	TGACG	697-701	Cis-acting element involved in the MeJA responsiveness
CoXTH25p	ABRE3a	TACGTG	1600-1605	Cis-acting element involved in the abscisic acid responsiveness
CoXTH25p	G-box	TACGTG	1600-1605	Cis-acting regulatory element involved in light responsiveness
CoXTH25p	ARE	AAACCA	119-124	Cis-acting regulatory element essential for the anaerobic induction

CoXTH25p	ARE	AAACCA	1038-1043	Cis-acting regulatory element essential for the anaerobic induction
CoXTH25p	ARE	AAACCA	1047-1052	Cis-acting regulatory element essential for the anaerobic induction
CoXTH25p	ARE	AAACCA	1073-1078	Cis-acting regulatory element essential for the anaerobic induction
CoXTH25p	ARE	AAACCA	1082-1087	Cis-acting regulatory element essential for the anaerobic induction
CoXTH25p	ARE	AAACCA	1210-1215	Cis-acting regulatory element essential for the anaerobic induction
CoXTH25p	TGACG-motif	TGACG	697-701	Cis-acting regulatory element involved in the MeJA responsiveness
CoXTH25p	CGTCA-motif	CGTCA	1454-1458	Cis-acting regulatory element involved in the MeJA responsiveness
CoXTH25p	MYB	TAACCA	1159-1164	MYB binding site involved in drought-inducibility
CoXTH25p	MYB	TAACCA	1960-1965	MYB binding site involved in drought-inducibility
CoXTH25p	CCGTCC-box	CCGTCC	1404-1409	Cis-acting regulatory element related to fruit development
CoXTH25p	CCGTCC-box	CCGTCC	1467-1472	Cis-acting regulatory element related to fruit development
CoXTH25p	MYB	TAACCA	1159-1164	MYB binding site involved in drought-inducibility
CoXTH25p	MYB	CAACCA	1442-1447	MYB binding site involved in drought-inducibility
CoXTH25p	MYB	TAACCA	1960-1965	MYB binding site involved in drought-inducibility
CoXTH25p	AAGAA-motif	GAAAGAA	913-919	Cis-acting element involved in the abscisic acid responsiveness
CoXTH25p	AAGAA-motif	GAAAGAA	1548-1554	Cis-acting element involved in the abscisic acid responsiveness
CoXTH25p	ABRE	ACGTG	1601-1605	Cis-acting element involved in the abscisic acid responsiveness
CoXTH26p	CAT-box	GCCACT	762-767	Cis-acting regulatory element related to meristem expression
CoXTH26p	GC-motif	CCCCCG	641-646	Enhancer-like element involved in anoxic specific inducibility
CoXTH26p	G-Box	CACGTG	970-975	Cis-acting regulatory element involved in light responsiveness
CoXTH26p	ABRE	ACGTG	739-743	Cis-acting element involved in the abscisic acid responsiveness
CoXTH26p	ABRE	CACGTG	970-975	Cis-acting element involved in the abscisic acid responsiveness
CoXTH26p	ABRE	ACGTG	971-975	Cis-acting element involved in the abscisic acid responsiveness
CoXTH26p	GA-motif	ATAGATAA	1668-1675	Part of a light responsive element
CoXTH26p	ARE	AAACCA	1994-1999	Cis-acting regulatory element essential for the anaerobic induction
CoXTH26p	ABRE3a	TACGTG	738-743	Cis-acting element involved in the abscisic acid responsiveness

CoXTH26p	MYB	CAACAG	397-402	MYB binding site involved in drought-inducibility
CoXTH26p	MYB	CAACCA	1943-1948	MYB binding site involved in drought-inducibility
CoXTH26p	MYB	CAACAG	397-402	MYB binding site involved in drought-inducibility
CoXTH26p	ABRE2	CCACGTGG	969-976	Cis-acting element involved in the abscisic acid responsiveness
CoXTH26p	Sp1	GGGCGG	216-221	Light responsive element
CoXTH26p	Sp1	GGGCGG	975-980	Light responsive element
CoXTH26p	ERE	ATTTTAAA	1365-1372	Ethylene response element
CoXTH26p	GT1-motif	GGTTAA	1526-1531	Light responsive element
CoXTH26p	W box	TTGACC	1692-1697	Stress-responsive element
CoXTH26p	MYC	CATTTG	596-601	MYC binding site
CoXTH26p	CCAAT-box	CAACGG	71-76	MYBHv1 binding site
CoXTH26p	CCAAT-box	CAACGG	189-194	MYBHv1 binding site
CoXTH26p	CCAAT-box	CAACGG	307-312	MYBHv1 binding site
CoXTH26p	CCAAT-box	CAACGG	716-721	MYBHv1 binding site
CoXTH26p	CCAAT-box	CAACGG	839-844	MYBHv1 binding site
CoXTH26p	CCAAT-box	CAACGG	1287-1292	MYBHv1 binding site
CoXTH26p	G-box	TACGTG	738-743	Cis-acting regulatory element involved in light responsiveness
CoXTH26p	G-box	CACGTG	970-975	Cis-acting regulatory element involved in light responsiveness
CoXTH26p	G-box	TAACACGTAG	1626-1634	Cis-acting regulatory element involved in light responsiveness
CoXTH26p	ABRE4	CACGTA	1629-1634	Cis-acting element involved in the abscisic acid responsiveness
CoXTH26p	Box 4	ATTAAT	28-33	Part of a conserved DNA module involved in light responsiveness
CoXTH26p	Box 4	ATTAAT	796-801	Part of a conserved DNA module involved in light responsiveness
CoXTH27p	GT1-motif	GGTTAA	357-362	Light responsive element
CoXTH27p	MYB	TAACCA	1790-1795	MYB binding site involved in drought-inducibility
CoXTH27p	DRE core	GCCGAC	1211-1216	Dehydration response element TC-rich repeats
CoXTH27p	I-box	AAGATAAGGCT	1402-1411	Part of a light responsive element

CoXTH27p	G-box	ACACGTGGC	1194-1202	Cis-acting regulatory element involved in light responsiveness
CoXTH27p	ABRE	GACACGTGGC	1193-1201	Cis-acting element involved in the abscisic acid responsiveness
CoXTH27p	ABRE	ACGTG	1196-1200	Cis-acting element involved in the abscisic acid responsiveness
CoXTH27p	ABRE	ACGTG	1215-1219	Cis-acting element involved in the abscisic acid responsiveness
CoXTH27p	ABRE	GCAACGTGTC	1231-1239	Cis-acting element involved in the abscisic acid responsiveness
CoXTH27p	ABRE	ACGTG	1234-1238	Cis-acting element involved in the abscisic acid responsiveness
CoXTH27p	GATA-motif	AAGATAAGATT	1402-1411	Part of a light responsive element
CoXTH27p	TCT-motif	TCTTAC	29-34	Part of a light responsive element
CoXTH27p	MYB	CAACAG	63-68	MYB binding site involved in drought-inducibility
CoXTH27p	MYB	CAACAG	293-298	MYB binding site involved in drought-inducibility
CoXTH27p	MYB	CAACAG	772-777	MYB binding site involved in drought-inducibility
CoXTH27p	ARE	AAACCA	399-404	Cis-acting regulatory element essential for the anaerobic induction
CoXTH27p	ARE	AAACCA	970-975	Cis-acting regulatory element essential for the anaerobic induction
CoXTH27p	ARE	AAACCA	1825-1830	Cis-acting regulatory element essential for the anaerobic induction
CoXTH27p	MYB	TAACTG	1039-1044	MYB binding site involved in drought-inducibility
CoXTH27p	CCAAT-box	CAACGG	338-343	MYBHv1 binding site
CoXTH27p	Box 4	ATTAAT	963-968	Part of a conserved DNA module involved in light responsiveness
CoXTH27p	WRE3	CCACCT	914-919	Wound responsiveness
CoXTH27p	MYC	CATTTG	1773-1778	MYC binding site
CoXTH27p	MYC	CATTTG	1924-1929	MYC binding site
CoXTH27p	GC-motif	CCCCCG	1619-1624	Enhancer-like element involved in anoxic specific inducibility
CoXTH27p	AAGAA-motif	GGTAAAGAAA	1398-1406	Cis-acting element involved in the abscisic acid responsiveness
CoXTH27p	MYB	CAACAG	63-68	MYB binding site involved in drought-inducibility
CoXTH27p	MYB	CAACAG	293-298	MYB binding site involved in drought-inducibility
CoXTH27p	MYB	CAACAG	772-777	MYB binding site involved in drought-inducibility
CoXTH27p	MYB	TAACCA	1790-1795	MYB binding site involved in drought-inducibility

CoXTH27p	STRE	AGGGG	1095-1099	Osmotic stress response element
CoXTH27p	STRE	AGGGG	1158-1162	Osmotic stress response element
CoXTH27p	circadian	CAAAGATATC	1286-1294	Cis-acting regulatory element involved in circadian control
CoXTH27p	circadian	CAAAGATATC	1881-1889	Cis-acting regulatory element involved in circadian control
CoXTH27p	O2-site	GATGATGTGG	325-333	Cis-acting regulatory element involved in zein metabolism regulation
CoXTH28p	chs-CMA1a	TTACTTAA	1767-1774	Part of a light responsive element
CoXTH28p	MYC	CATTTG	282-287	MYC binding site
CoXTH28p	MYC	CATTTG	940-945	MYC binding site
CoXTH28p	MYC	CATTTG	993-998	MYC binding site
CoXTH28p	ATCT-motif	AATCTAATCC	1644-1652	Part of a conserved DNA module involved in light responsiveness
CoXTH28p	I-box	gGATAAGGTG	297-305	Part of a light responsive element
CoXTH28p	ERE	ATTTTAAA	1326-1333	Ethylene response element
CoXTH28p	Box 4	ATTAAT	899-904	Part of a conserved DNA module involved in light responsiveness
CoXTH28p	W box	TTGACC	1753-1758	Stress-responsive element
CoXTH28p	DRE core	GCCGAC	11-16	Dehydration response element TC-rich repeats
CoXTH28p	WRE3	CCACCT	1184-1189	Wound responsiveness
CoXTH29p	MYB	CAACTG	1749-1754	MYB binding site involved in drought-inducibility
CoXTH29p	MRE	AACCTAA	1239-1245	MYB binding site involved in light responsiveness
CoXTH29p	LTR	CCGAAA	282-287	Cis-acting element involved in low-temperature responsiveness
CoXTH29p	ARE	AAACCA	1861-1866	Cis-acting regulatory element essential for the anaerobic induction
CoXTH29p	Box 4	ATTAAT	37-42	Part of a conserved DNA module involved in light responsiveness
CoXTH29p	CAT-box	GCCACT	1084-1089	Cis-acting regulatory element related to meristem expression
CoXTH29p	CAT-box	GCCACT	1201-1206	Cis-acting regulatory element related to meristem expression
CoXTH29p	GA-motif	ATAGATAA	671-678	Part of a light responsive element
CoXTH29p	MYB	TAACCA	1856-1861	MYB binding site involved in drought-inducibility
CoXTH29p	MYB	TAACCA	1856-1861	MYB binding site involved in drought-inducibility

CoXTH29p	MYC	CATTTG	426-431	MYC binding site
CoXTH29p	MBS	CAACTG	1749-1754	MYB binding site involved in drought-inducibility
CoXTH29p	WUN-motif	TTATTACAT	1838-1846	Stress-responsive element
CoXTH30p	GT1-motif	GGTTAAT	17-23	Light responsive element
CoXTH30p	GT1-motif	GGTTAA	1450-1455	Light responsive element
CoXTH30p	GT1-motif	GGTTAA	1993-1998	Light responsive element
CoXTH30p	MRE	AACCTAA	1499-1505	MYB binding site involved in light responsiveness
CoXTH30p	MYC	CATTTG	640-645	MYC binding site
CoXTH30p	G-box	CACGTC	744-749	Cis-acting regulatory element involved in light responsiveness
CoXTH30p	G-box	CACGTC	885-890	Cis-acting regulatory element involved in light responsiveness
CoXTH30p	I-box	TGATAATGT	194-202	Part of a light responsive element
CoXTH30p	ABRE4	CACGTA	102-107	Cis-acting element involved in the abscisic acid responsiveness
CoXTH30p	MYB	TAACCA	719-724	MYB binding site involved in drought-inducibility
CoXTH30p	MYB	TAACCA	1462-1467	MYB binding site involved in drought-inducibility
CoXTH30p	ERE	ATTTCATA	447-454	Ethylene response element
CoXTH30p	ERE	ATTTTAAA	1203-1210	Ethylene response element
CoXTH30p	ERE	ATTTTAAA	1242-1249	Ethylene response element
CoXTH30p	ARE	AAACCA	1332-1337	Cis-acting regulatory element essential for the anaerobic induction
CoXTH30p	MYB	TAACCA	719-724	MYB binding site involved in drought-inducibility
CoXTH30p	MYB	TAACCA	1462-1467	MYB binding site involved in drought-inducibility
CoXTH30p	O2-site	GATGATGTGG	195-203	Cis-acting regulatory element involved in zein metabolism regulation
CoXTH30p	AE-box	AGAAACAA	1347-1354	Part of a module for light response
CoXTH31p	MYC	CAATTG	118-123	MYC binding site
CoXTH31p	MBS	CAACTG	1200-1205	MYB binding site involved in drought-inducibility
CoXTH31p	ERE	ATTTTAAA	61-68	Ethylene response element
CoXTH31p	ERE	ATTTTAAA	1523-1530	Ethylene response element

CoXTH31p	AAGAA-motif	GAAAGAA	1205-1211	Cis-acting element involved in the abscisic acid responsiveness
CoXTH31p	Box 4	ATTAAT	664-669	Part of a conserved DNA module involved in light responsiveness
CoXTH31p	Box 4	ATTAAT	751-756	Part of a conserved DNA module involved in light responsiveness
CoXTH31p	Box 4	ATTAAT	755-760	Part of a conserved DNA module involved in light responsiveness
CoXTH31p	MYB	CAACCA	519-524	MYB binding site involved in drought-inducibility
CoXTH31p	MYB	CAACAG	1858-1863	MYB binding site involved in drought-inducibility
CoXTH31p	O2-site	GATGATGTGG	1247-1255	Cis-acting regulatory element involved in zein metabolism regulation
CoXTH31p	TCCC-motif	TCTCCCT	1877-1883	Part of a light responsive element
CoXTH31p	TGACG-motif	TGACG	780-784	Cis-acting regulatory element involved in the MeJA responsiveness
CoXTH31p	as-1	TGACG	780-784	Cis-acting, element involved in the MeJA responsiveness
CoXTH31p	MYB	CAACTG	1200-1205	MYB binding site involved in drought-inducibility
CoXTH31p	ABRE	ACGTG	383-387	Cis-acting element involved in the abscisic acid responsiveness
CoXTH31p	MYC	TCTCTTA	349-355	MYC binding site
CoXTH31p	MYB	CAACAG	1858-1863	MYB binding site involved in drought-inducibility
CoXTH31p	ARE	AAACCA	846-851	Cis-acting regulatory element essential for the anaerobic induction

Table S4. Primers used in the study

Primer name	Squences (5' to 3')
**For qRT-PCR**	
GAPDH-qF	CTACTGGAGTTTTCACCGA
GAPDH-qR	TAAGACCCTCAACAATGCC
CoXTH1-qF	ACACGAGCATAGTTGGTGCT
CoXTH1-qR	CAGCAGAGTTACCAGGCACA
CoXTH2-qF	CCTCTCCGTTTCAGGACGAC
CoXTH2-qR	CGGCTGTCCTGTCCTATTCC
CoXTH3-qF	GGCGTTCCATACCCGAAGAA
CoXTH3-qR	CAACCGCAAGAAGTTGCTCC
CoXTH4-qF	TCTTCCGGCAACTTCTACCG
CoXTH4-qR	GAAGAGTTTCCGGGGACGAG
CoXTH5-qF	AGGTTGTGGTTTTGCCTCCA
CoXTH5-qR	CCTCTGCTCCCTATCACCCT
CoXTH6-qF	TGGGTGGAGACTTCCCTTCA
CoXTH6-qR	ACACGAGCATAGTTGGTGCT
CoXTH7-qF	CCATGCCTCAGTTCTTGACCA
CoXTH7-qR	GAATGAGCCCCCGATGTTGA
CoXTH8-qF	GCCAATGTGGGTCTATGGGT
CoXTH8-qR	ATGTGTTGCCCTCTGGTGAG
CoXTH9-qF	TGGCTTCCCAACACAACCAA
CoXTH9-qR	CGAACTCCACCAACAGCAAC
CoXTH10-qF	GGCTTATCCACCGGCTCATT
CoXTH10-qR	GAAGTGTCGGTTCCAGCAGA
CoXTH11-qF	GTGAATCAATCGGCGTCCCT
CoXTH11-qR	CCATGCCCCACTCGAAAATG
CoXTH12-qF	GTGCAGCCATCTCCTCTGTT
CoXTH12-qR	TGCCCATTCCAACCCATTGA
CoXTH13-qF	TTGCGCTCGGAATTGGAAAC
CoXTH13-qR	TTGTCGCCCAGTTATCAGCA
CoXTH14-qF	GCAAAGGCAACAGAGAGCAA
CoXTH14-qR	GTCTTCACAAGGCCACCTCT
CoXTH15-qF	ATGCCGATAACTGGGCAACA
CoXTH15-qR	CCTCTGGAAGTCGGTGCAAT
CoXTH16-qF	GGGTGTTCCATTCCCCACAA
CoXTH16-qR	TGTACTTGCGCTGTACCCAT
CoXTH17-qF	AGCTCCTTTCATTGCCTGCT
CoXTH17-qR	AACACACATGCTCCCAGAGG
CoXTH18-qF	AGCTCGATTTGGAACGCAGA
CoXTH18-qR	TTGGCTCGTCCCACCAATAC
CoXTH19-qF	CGTGCCAGAATACGCAATGG
CoXTH19-qR	TGCCCACTAACATTCCCCAA
CoXTH20-qF	GAGGCACCAGCTTTGGTTTG

CoXTH20-qR	CCTCCTCTTGTTGCCCAGTT
CoXTH21-qF	GCCATTGCACGCCATAGTTT
CoXTH21-qR	GGTTGGCCCTTCCGATGATA
CoXTH22-qF	CAGCATACTACCGGGGCTTC
CoXTH22-qR	ACGTTTGCACTCTGGAGGAA
CoXTH23-qF	GGCTTTGCCATTGACGGATG
CoXTH23-qR	GGGGGATATGTTGGGCGTTT
CoXTH24-qF	CCCAGGAAGCCTGTTGATGT
CoXTH24-qR	TCTCATCATGCTCCGCGTTT
CoXTH25-qF	GACAGTTCATCGGGGTCTGG
CoXTH25-qR	ACCAGAGATGGAGCCTTTGC
CoXTH26-qF	GGACTTTCCATCCAAGCCCA
CoXTH26-qR	ATTCGGGGCATGGTCACATT
CoXTH27-qF	TCGCGATGAGATGGACTTCG
CoXTH27-qR	CACTCCCTTGTCTGCATGGT
CoXTH28-qF	ACCTATGGGGTCCTCAGCAT
CoXTH28-qR	GCTTATCCGGTGTCGTTCCA
CoXTH29-qF	TCGGCGTTCTTGCCATACAT
CoXTH29-qR	ATAGAAGGCCACCACTGTGC
CoXTH30-qF	AAGATTGACTGGAGCTGGGC
CoXTH30-qR	GCATCTGTCCGTTGAATCGC
CoXTH31-qF	CTACTCGCCTCTCTTCAGCG
CoXTH31-qR	CGTCTGGAACCTCCATGGTC
Atactin11-qF	CCCGCTATGTATGTCGCCA
Atactin11-qR	AACCCTCGTAGATTGGCACA
**For cloning and vector construction**	
pYES-*CoXTH1*-F	cttggtaccgagctcggatccATGAATTCTCAAACTTTATTTTCTAAAAAG
pYES-*CoXTH1*-R	tgatggatatctgcagaattcTCATGTTAGTGAGCATTCTTGAGG
pYES-*CoXTH6*-F	cttggtaccgagctcggatccATGGAAACTTTTCATTCTCTCTCC
pYES-*CoXTH6*-R	tgatggatatctgcagaattcTCAATCTTCATGCTGGTCTCC
pYES-*CoXTH14*-F	cttggtaccgagctcggatccATGGCTTCTTCTTCTTCTAGCTCAG
pYES-*CoXTH14*-R	tgatggatatctgcagaattcTTATGAGATGCTGCATTCCGG
pYES-*CoXTH28*-F	cttggtaccgagctcggatccATGTTCCCTTCTTGCAATGCTG
pYES-*CoXTH28*-R	tgatggatatctgcagaattcCTAACACTCTGGTATTTGGTTATGGTC
pYES-*CoXTH31*-F	cttggtaccgagctcggatccATGGGCTGTTTACGTTCTCTCTCC
pYES-*CoXTH31*-R	tgatggatatctgcagaattcTCACATATCAGAAGCAGCCTGG
pBI-*CoXTH1*-F	acgggggactctagaggatccATGAATTCTCAAACTTTATTTTCTAAAAAGA
pBI-*CoXTH1*-R	cgatcggggaaattcgagctcTCATGTTAGTGAGCATTCTTGAGGC

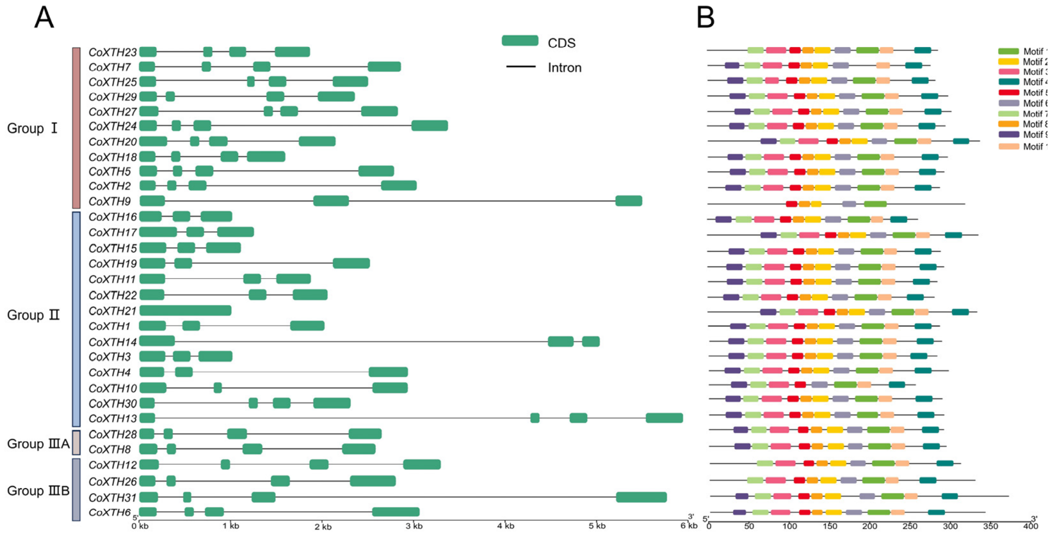

Figure S1. Gene structure and conserved motifs of *CoXTH* family genes. A, Gene structure, The green boxes indicate coding sequences (CDS), and line segments represent intron sequences; B, The conserved motifs of *CoXTH* family genes. Different colored boxes represent different conserved motifs

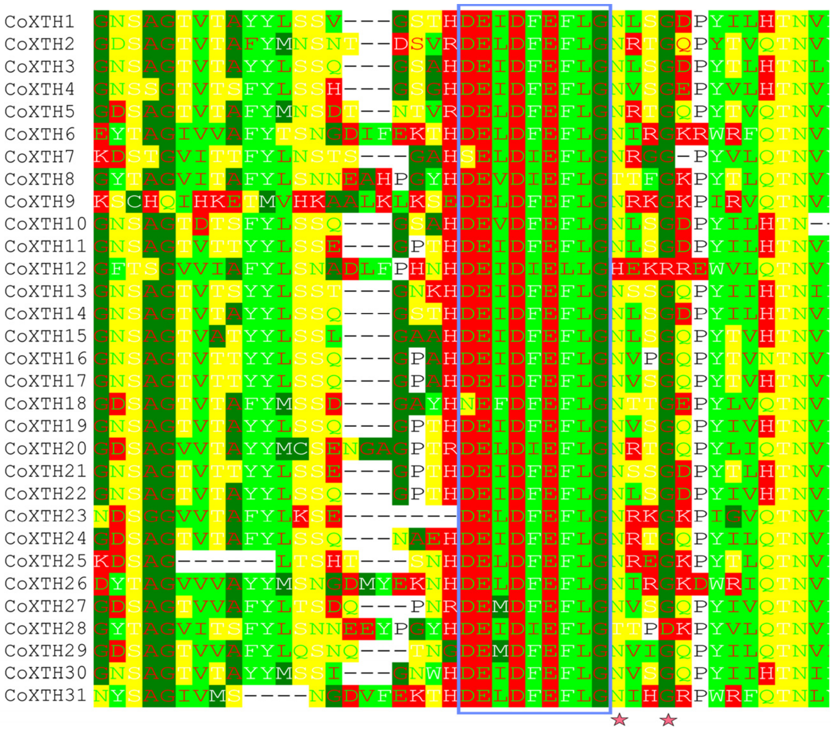

Figure S2. The active catalytic regions of CoXTH protein family. The rectangular frames represent active catalytic regions, and the asterisks represent N-linked glycosylation sites.

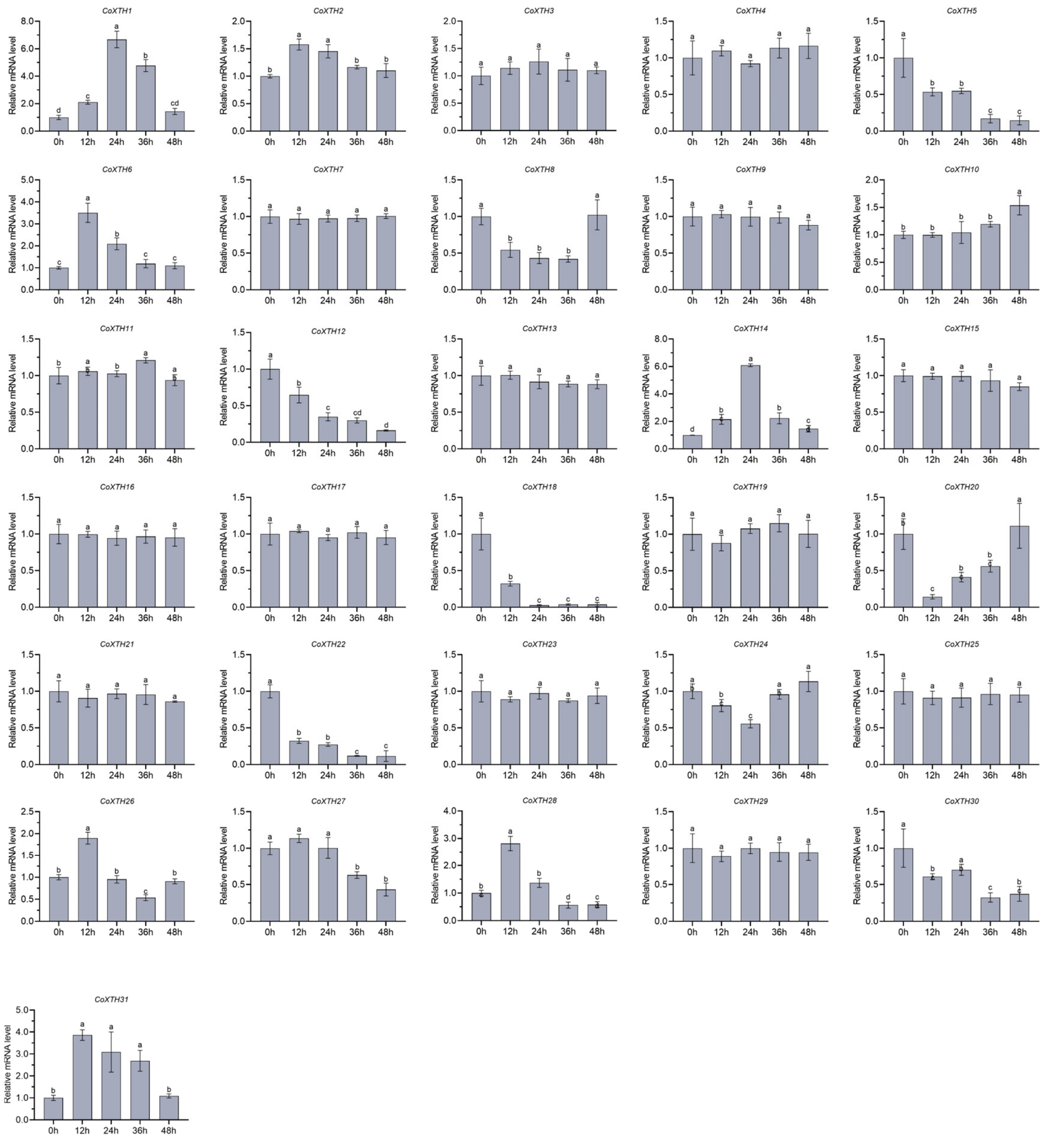

Figure S3. RT- qPCR analysis of the *CoXTH* genes under drought stress. The data are expressed as mean ± SD (n =3). Different letters indicate significant differences at $p < 0.05$.

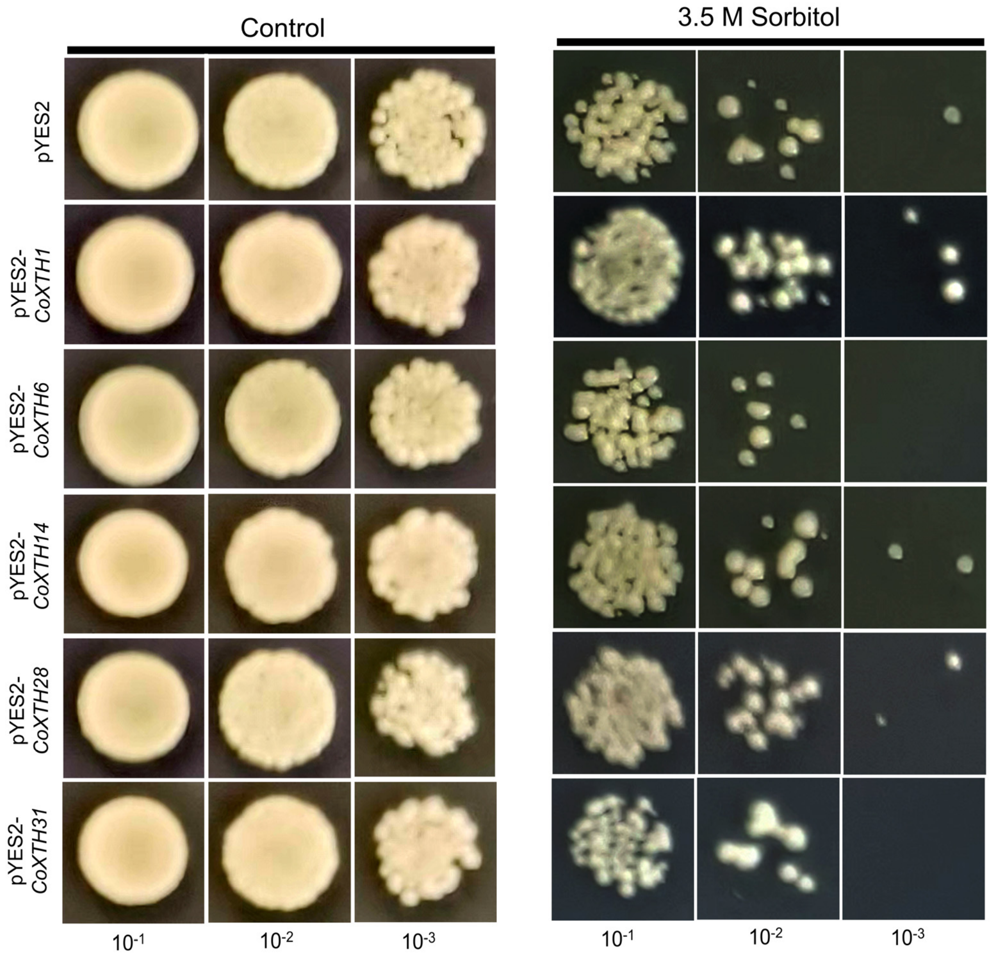

Figure S4. Comparison of the growths of osmotolerance yeast cells. Heterologous expression of *CoXTH1*, *CoXTH6*, *CoXTH14*, *CoXTH28*, and *CoXTH31*. The heterologously expressed yeast strains pYES2, pYES2-*CoXTH1*, pYES2-*CoXTH6*, pYES2-*CoXTH14*, pYES2-*CoXTH28*, and pYES2-*CoXTH31* were grown on SG/-Ura media containing 3.5 M sorbitol for 24 h, after spotting onto SD/-Ura media for 3 days.

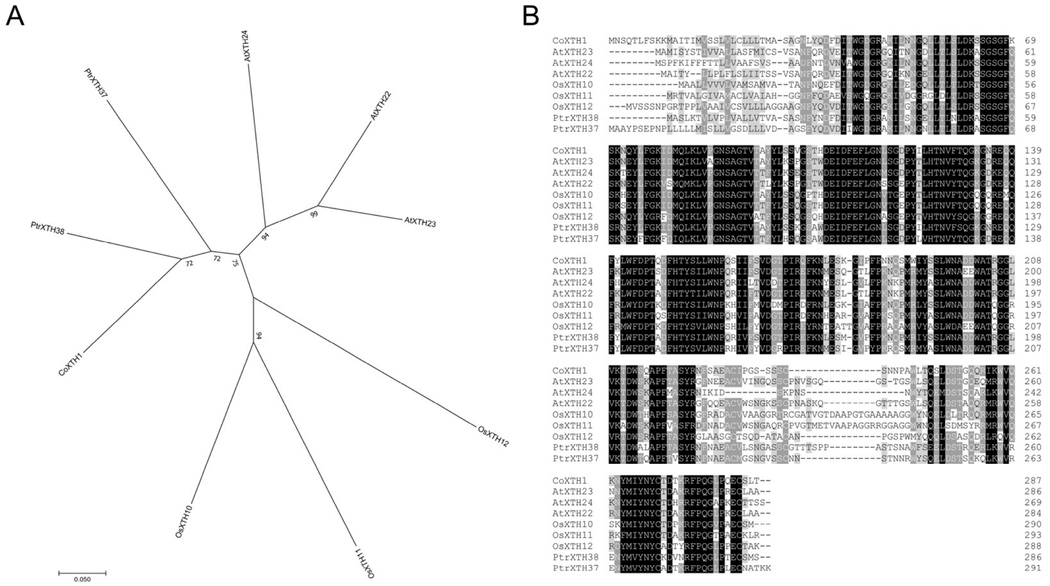

Figure S5. Sequence analysis of CoXTH1. A, Phylogenetic tree of CoXTH1 and its homolog proteins in *O. sativa*, *A. thaliana*, and *P. trichocarpa*. The phylogenetic tree was generated using the neighbor-joining (NJ) method, with 1000 bootstrap replicates. B, Multiple comparisons of CoXTH1 and its homologs in *O. sativa* and *A. thaliana*. AtXTH22 (At5g57560), AtXTH23 (At4g25810), AtXTH24 (At4g30270), OsXTH10 (Os06t0697000), OsXTH11(Os06g48160), OsXTH12 (Os06g48180), PtrXTH37 (Potri.018G094900), PtrXTH38 (Potri.018G095100).

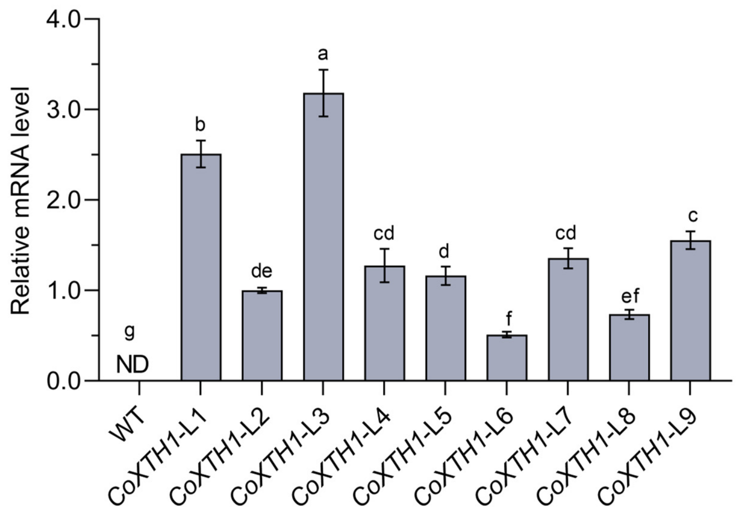

Figure S6. RT-qPCR analysis of the *CoXTH1* gene in different *A. thaliana* transgenic lines. The data are expressed as mean ±SD (n =3). The data are expressed as mean ± SD (n =3). Different letters indicate significant differences at $p < 0.05$.

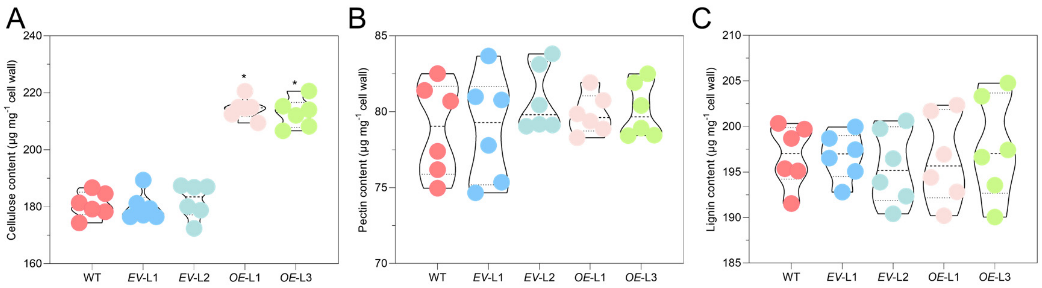

Figure S7. The content of cellulose (A), pectin (B), and lignin(C) in *A. thaliana*. WT, *EV*, and *OE* seedlings were grown on 1/2 MS media with 0, 75 mM mannitol for 10 d. The data are expressed as mean ± SD (n =6). Different letters show significant differences at $p < 0.05$.
